# Lifestyle in Emerging Adults with Type 1 Diabetes Mellitus: A Qualitative Systematic Review

**DOI:** 10.3390/healthcare12030309

**Published:** 2024-01-25

**Authors:** María-Ángeles Núñez-Baila, Anjhara Gómez-Aragón, Armando-Manuel Marques-Silva, José Rafael González-López

**Affiliations:** 1 Nursing Department, Faculty of Nursing, Physiotherapy and Podiatry, Universidad de Sevilla, 41009 Seville, Spain; mnbaila@us.es (M.-Á.N.-B.); joserafael@us.es (J.R.G.-L.); 2 Department of Nursing, Escola Superior de Enfermagem de Coimbra, 3004-011 Coimbra, Portugal; armandos@esenfc.pt; 3 Unidade de Investigação em Ciências da Saúde: Enfermagem (UICISA: E), 3004-011 Coimbra, Portugal

**Keywords:** adult, emerging adult, lifestyle, young adult, type 1 diabetes

## Abstract

Emerging adulthood is a transitional stage with significant lifestyle changes, making it especially challenging for those living with type 1 diabetes mellitus. This systematic review synthesizes qualitative research to explore how emerging adulthood (18–29 years) influences lifestyle behaviors in individuals with type 1 diabetes mellitus. CINAHL, Cochrane Library, Global Health, Nursing & Allied Health Premium, PsycINFO, PubMed, Scopus, and WOS were searched for original qualitative studies addressing the lifestyle of 18–31-year-olds with type 1 diabetes mellitus, published between January 2010 and March 2021 following the Preferred Reporting Items for Systematic Reviews and Meta-Analyses guidelines. Thirty-five studies met the inclusion criteria and their findings were categorized into eight topics (emotions and feelings, nutrition, perceptions, risky behaviors, self-care, sleep, social relationships, and stigma) using meta-aggregation, as outlined in the *Joanna Briggs Institute Manual for Evidence Synthesis*. The spontaneity characteristic of emerging adulthood can undermine self-care. This is because new environments, schedules, and relationships encountered during this life stage often lead to the neglect of diabetes management, owing to the various social, academic, and occupational demands. This review highlights the necessity of creating health promotion strategies tailored to the unique lifestyle aspects of emerging adults with type 1 diabetes mellitus.

## 1. Introduction

Throughout emerging adulthood, a stage defined by Arnett [1] and covering ages 18 to 29, most of the legally adult people do not fully embrace adult identity due to factors such as persistent economic dependence, extended educational engagement, and challenges in accessing or securing stable employment [2]. This situation results in a delay in achieving traditional adult social milestones and, thereby, conventional adult roles [3,4]. Additionally, this stage was characterized by a great deal of exploration in human spheres and activities such as sexuality, physical exercise, nutrition, socio-occupational relationships, sleep patterns, self-care, and, in many cases, the consumption of alcohol, tobacco, or other drugs [5,6,7]. While adolescence is also a time of self-discovery and experimentation, it differs primarily in the fact that adolescents are not legally considered adults and they have a lower spending power [8].

For emerging adults, managing chronic diseases like type 1 diabetes mellitus (T1DM) can be particularly disruptive. The required attention often collides with external social demands such as friendships, partnerships, studies, and the overall pace of life [9,10]. According to the International Diabetes Federation the global prevalence of T1DM, which is increasing every year, was 8.75 million [11]. The onset of T1DM usually occurred in childhood or adolescence, which are the most investigated stages [12]. However, recent data indicated that 62% of new T1DM diagnoses occurred in individuals over 20, highlighting a shift in research focus to adult life stages [11]. The multidimensional treatment of T1DM involves medication management, blood glucose monitoring, carbohydrate counting, and physical exercise [9]. Consequently, the impact of day-to-day diabetes management becomes unavoidable and may lead to lifestyle discordances [10].

Within the context of emerging adulthood and chronic illness management, Meleis’ transition theory offers a pivotal framework [13]. This theory underscores the significant changes and transitional periods individuals experience, crucial to their development. According to Meleis, these transitions involve shifts not only in health and illness but also in identity, roles, and relationships [13]. For emerging adults with T1DM, these transitions are particularly profound, requiring navigation through the complexities of their chronic condition alongside the life transitions characteristic of this stage [9]. Understanding these processes is vital for developing tailored care strategies and support systems, aimed at addressing the specific needs of these individuals [14].

T1DM demands constant self-management and adherence, potentially leading to emotional stress and distress due to the individual’s active role in required self-care [15,16]. For this very reason, it is necessary to know how self-care practices are integrated into the rest of the activities which form their lifestyle, such as nutrition, physical activity, sleep, sexuality, social relationships, and risky behaviors such as alcohol consumption. At the same time, it is also necessary to understand the similitudes of these behaviors with respect to those of their peers without T1DM. A qualitative methodology allows for an in-depth understanding of the reasons for adopting certain behaviors. Several authors [12,17,18] claimed that using an exclusively quantitative methodology in the study of lifestyle leads to a lack of knowledge about the motivations behind the behaviors adopted. Therefore, the objective of this systematic review is to comprehensively examine the various lifestyle aspects of emerging adults with T1DM, recognizing that this condition may necessitate unique considerations in different life spheres during a challenging, transitional phase marked by significant changes and demands. By analyzing qualitative studies, this review aims to uncover the underlying motivations for behaviors in emerging adults with T1DM and identify significant gaps, thereby guiding future research directions in this domain. This approach aims to facilitate a deeper understanding of how uniquely T1DM impacts lifestyle choices and challenges during this critical developmental stage.

## 2. Materials and Methods

This systematic review analyzes, through qualitative studies, the influence of emerging adulthood stage on the adoption of lifestyle behaviors in individuals with T1DM. Consequently, the guiding question of this review is as follows: How does emerging adulthood influence the lifestyle choices and challenges of individuals with type 1 diabetes mellitus?

This qualitative systematic review was conducted in accordance with the Preferred Reporting Items for Systematic Reviews and Meta-Analyses (PRISMA) [19]. In addition, it has a protocol registered in the international prospective register of systematic reviews PROSPERO, with identification CRD42021240637.

### 2.1. Search Strategy

Regarding terminology, the Medical Subject Headings (MeSH) and Health Sciences Descriptors (DeCS) thesauri provided two terms for defining the life stage following adolescence. The term ‘Adult’, introduced in 1996, covered a wide age range, from 19 to 44 years, reflecting a period with varied characteristics. In 2009, the term ‘Young Adult’ was added to specifically refer to individuals aged 19 to 24 years. However, this review, aligning with Arnett’s research [1,3,5], established an age range of 18–29 years, and thus used ‘Emerging Adult’ in its title. Moreover, ‘Adult’ and ‘Young Adult’ were included as keywords for the search strategy to collectively cover the group aged 18–29, as previously indicated.

The systematic search, conducted from March to November 2021, encompassed several databases: CINAHL, Cochrane Library, Global Health, Nursing & Allied Health Premium, PsycINFO, PubMed, Scopus, and WOS. The uniform search strategy applied across these databases included the following terms: (adult OR “young adult”) AND (“type 1 diabetes”) AND (lifestyle OR behavior OR health OR “health risk behavior*”).

The search was structured in accordance with the PI(E)CO framework:

- Participants: Young adults (18 to 29 years old) with T1DM or insulin-dependent diabetes.
- Intervention/Exposure: Behaviors adopted by these young adults in their lifestyle.
- Context (instead of Comparison): Age stage of young adults (18 to 29 years old).
- Outcomes to be measured: Specific characteristics of the different lifestyle dimensions (nutrition, sleep, physical exercise, social, work and environmental relationships, risky behavior, and self-care).

### 2.2. Eligibility Criteria

The eligibility criteria for study inclusion were as follows:

- They were primary qualitative research or mixed methods studies with a significant and explicitly stated qualitative component, focusing on lifestyle aspects and demonstrating consistent methodological quality. Studies had to be available in full text and published between 1 January 2010 and 31 March 2021.
- The participant sample needed to consist of emerging adults, defined as individuals aged 18–29 years according to Arnett’s broadest definition [1,3,5]. To encompass a wider range of relevant studies, the age criterion was flexibly extended by +2 years through consensus among the authors. Therefore, the final sample of the selected studies included individuals aged 18–31 years.
- Studies were considered if written in English, Spanish, or Portuguese.

Studies using exclusively quantitative methodology or not related to the object of this review were excluded. In addition, other types of texts were excluded: commentaries, clinical guidelines, dissertations, conference abstracts, editorial letters, PhD theses, books, chapter books, reports, and systematic reviews with or without meta-analyses.

### 2.3. Quality Appraisal

The methodological quality of the selected articles was evaluated using the Joanna Briggs Institute (JBI) Critical Appraisal Checklist for Qualitative Research [20]. Inclusion criteria required that studies showed at least 80% of positive aspects in this assessment, indicative of a high methodological quality. This evaluation was primarily conducted by the first reviewer. In instances where scores were marginally close to the minimum threshold and there was uncertainty regarding inclusion, the assessments were further reviewed and corroborated by the second and third reviewers. In short, of the 34 articles assessed, 21 articles (61.76%) achieved a score of 80%, 11 articles (32.35%) scored 90%, and 2 articles (5.88%) attained a score of 100%. The most common factors leading to lower scores were the absence of explicit details regarding the cultural and theoretical position of the researcher, as well as the lack of clarity on the mutual influence between the researcher and the research.

### 2.4. Bias in the Review Process

Several measures were implemented to mitigate biases in the review process. To address publication bias, duplicate studies were identified and removed. Selection bias was minimized by defining inclusion criteria prior to the search and conducting a peer review process in accordance with PRISMA recommendations. To counter study quality and report bias, the Joanna Briggs Institute (JBI) Critical Appraisal Checklist for Qualitative Research was employed, as this tool ensures the inclusion of studies that demonstrate high-quality evidence and adhere to correct qualitative methodologies [20]. The use of the JBI checklist helped to mitigate publication bias by providing a standardized framework for evaluating the rigor and credibility of the qualitative research, ensuring that only studies meeting these criteria were included in the review [21].

### 2.5. Study Selection and Data Extraction Process

In the initial phase of study selection, the first reviewer conducted a systematic screening of titles and abstracts based on predefined inclusion and exclusion criteria. This was followed by an exhaustive full-text review of the articles preliminarily selected to verify their compliance with the established criteria. Subsequently, these full-text reviewed articles underwent an independent evaluation by a second reviewer. The reasons for exclusions, both from the initial discard by the first reviewer and the subsequent exclusions by the second reviewer, were meticulously documented following this comprehensive full-text review.

Concurrently, the process of data extraction and synthesis was conducted with scrupulous attention to detail. Key data extracted from the studies—encompassing publication year, geographical location, authorship, study title, sample demographics (age and sex), employed qualitative methodologies, ethics committee approvals, and the nature of participant discourses (categorized as primary or secondary results), as well as lifestyle behaviors addressed—were systematically organized and recorded in a Microsoft Excel® v2016 database. This task of data extraction was independently undertaken by both the first and second reviewers.

### 2.6. Data Synthesis

The synthesis of data in this systematic review was conducted using the meta-aggregation approach, which is particularly suited for inclusive reviews of diverse qualitative evidence across various paradigms. This method aligns well with the specific requirements of qualitative research, facilitating a comprehensive understanding of the data. Unlike other qualitative synthesis methods, meta-aggregation allows for the preservation of the unique voices and perspectives within each study while facilitating a cohesive and comprehensive understanding of the data. This method is particularly adept at handling the rich, varied nature of qualitative research, making it an ideal fit for our study’s aim to explore the complex lifestyle dimensions of emerging adults with T1DM [22].

In this review, a ‘finding’ refers to any qualitative insight related to the lifestyle dimensions of emerging adults with T1DM, encompassing behaviors, experiences, and perceptions relevant to nutrition, sleep, physical exercise, social interactions, risky behavior, and self-care. The finding extraction process, guided by the PI(E)CO framework, involved systematically identifying these findings from each study, focusing on the nuanced behaviors and experiences of the target population. This rigorous approach ensures a detailed and comprehensive synthesis of qualitative evidence pertinent to the lifestyle management of emerging adults with T1DM.

Following the extraction of relevant findings, the thematic categorization of these data was primarily undertaken by the first author. This stage involved a detailed analysis where the extracted findings were grouped into the aforementioned initial categories as per the PI(E)CO framework. During this process, the second author played a critical role in reviewing and refining the categorization, bringing an additional layer of scrutiny to ensure that the thematic analysis was robust and comprehensive.

For the organization of data, our team used a shared document. This approach enabled efficient collaboration and discussion, allowing us to systematically compile, analyze, and refine our findings without specialized software. In instances of disagreement, detailed discussions were held to explore differing perspectives. The collaborative dialogue between all authors facilitated informed consensus, ensuring that each decision made reflected a comprehensive and shared understanding of the data. This consensus approach ensured that the synthesis of findings was coherent, balanced, and representative of all voices in the research team.

However, a deeper examination of the data by the research team led to the realization that additional categories were necessary to capture the full spectrum of influences on the behaviors of emerging adults with T1DM. Therefore, new categories such as emotions and feelings, perceptions (including self-perception and health perception), and stigma were introduced. These categories, though not strictly behaviors, encapsulate crucial psychosocial traits exerting a significant influence on the lifestyle choices of the target group.

The final step of synthesizing the identified thematic categories was a collaborative effort involving all authors. Both the second and third authors played a pivotal role in this phase, facilitating the integration and summarization of both the predefined and emergent themes. This crucial involvement ensured the construction of a cohesive and comprehensive understanding of the key lifestyle aspects and psychosocial influences on emerging adults with T1DM. Meanwhile, the fourth author provided continual support throughout the process, leveraging his extensive experience in systematic reviews as he was trained at the Joanna Briggs Institute. This expertise was instrumental in ensuring that the meta-aggregation process was meticulously conducted and aligned with best practices in qualitative synthesis. The results of the synthesis and meta-aggregation are detailed in the Supplementary Material, Table S1.

### 2.7. Confidence in the Findings: Application of CONQual

This systematic review applied the JBI’s CONQual approach to rigorously assess the confidence in the synthesized findings from qualitative studies [23].

Ranking Levels: In CONQual, findings are classified within a scale of ‘High’, ‘Moderate’, ‘Low’, and ‘Very Low’, reflecting their initial quality level. Qualitative studies typically start at ‘High’ due to their detailed methodology and depth of analysis.

Dependability Assessment: The dependability of each finding was thoroughly evaluated. A finding meeting 4–5 criteria for methodological congruence and researcher’s influence retained its ‘High’ rating. Findings with 2–3 criteria met were downgraded by one level, and those with 0–1 criterium were downgraded by two levels.

Credibility Assessment: The credibility of each finding was subsequently assessed. Findings were categorized as ‘Unequivocal’, ‘Credible’, or ‘Not Supported’, based on the evidence type and robustness. ‘Unequivocal’ findings maintained their original ranking, while ‘Credible’ findings were downgraded by one level, and mix of ‘Credible/Not Supported’ findings were downgraded by three levels.

This structured approach combines initial rankings with thorough dependability and credibility assessments, ensuring a comprehensive evaluation of the synthesized findings. It demonstrates the overall confidence that can be placed in them within our systematic review, clearly reflecting their dependability and credibility. In this systematic review, we classified 2 synthesized findings as ‘Low’, 22 as ‘Moderate’, and 10 as ‘High’. Consequently, we can conclude there is a high degree of confidence in the findings of this review. Further details are provided in Table S2.

## 3. Results

### 3.1. Search Outcomes

**Figure 1:**
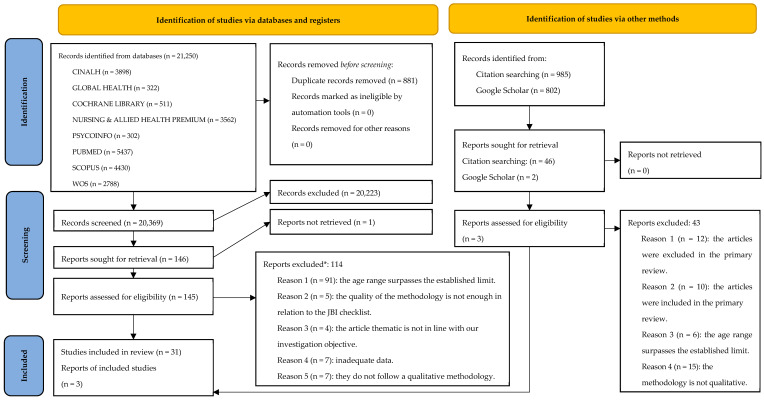
PRISMA flow. *Articles that fall outside the specified age range but which also met at least one other criterion were counted solely under the first reason.

An exhaustive search across eight databases yielded a total of 21,250 records. Following the application of specified inclusion and exclusion criteria and the elimination of duplicate entries, a total of 34 articles were selected for inclusion. This selection comprised 31 studies identified from the initial search and an additional 3 derived from citation tracking. The process of article selection is comprehensively detailed in the PRISMA flow diagram presented in Figure 1.

Of the articles initially identified, 114 were subsequently excluded from primary research consideration. The reasons for exclusion were as follows: 91 articles were eliminated due to their inclusion of sample populations exceeding the predetermined age range, 7 were excluded on the basis of inadequate data, 7 were ruled out for not adhering to a qualitative research methodology, 5 were dismissed for failing to meet the requisite methodological robustness as per the JBI checklist criteria, and 4 were excluded for lacking relevance to the central theme of this research.

### 3.2. Study Characteristics

The included studies are original qualitative research, employing methods such as in-depth or semi-structured interviews, focus groups, and qualitative surveys. The majority of participants were women, with two studies exclusively involving women [24,25].

Over half of the studies (n = 19) originated from the United States, with the remainder sourced from Australia, Canada, India, Ireland, Portugal, South Africa, Sweden, Turkey, and the United Kingdom. Table 1 outlines the characteristics of each study.

### 3.3. Data Synthesized Based on Categories

#### 3.3.1. Emotions and Feelings

In the stage of emerging adulthood, a complex amalgam of feelings and emotions associated with T1DM is observed [43,45]. This emotional complexity is attributed to the continuous care demands involved in managing this chronic disease [43,45,49]. Emotional responses such as ‘distress’, specifically termed ‘diabetes distress’ or ‘burnout’, are commonly identified. Among the most prevalent emotions and feelings are denial, frustration, fear, worry, boredom, fatigue, demotivation, indignation, loneliness, misunderstanding, guilt, shame, exhaustion, emotional tension, sense of invincibility, and shock [25,32,34,35,37,40,41,44,45,48,51].

This diversity of feelings arises during a critical transition phase, where emerging adults experience a shift of responsibilities from their parents to themselves [26,37]. In most cases, the management of T1DM falls entirely on these individuals [24,37,40,48]. This shift in responsibility, combined with the demands of adult life, such as academic and work challenges, as well as constant changes in routines and schedules typical of this stage, can create an imbalance between personal demands and available resources, leading to emotional imbalance [27,30,31,36,37,43,46,48,49,50,56]. Moreover, this life stage is often marked by an engagement in risky behaviors, such as alcohol consumption, which can pose significant challenges for individuals with T1DM [40,46,49,50]. Emerging adults frequently seek advice on managing these behaviors in conjunction with their condition from healthcare providers [28,47]. However, they often face barriers, such as a lack of trust or unrealistic advice that fails to align with their lifestyle and situational realities [32,33,41,47]. This disconnect not only pertains to risky behaviors but also extends to their overall lifestyle management, further complicating their emotional and psychosocial experience [34,40,47,49].

Furthermore, it is important to consider that the emergence of these emotions can be influenced by interactions with the environment [24,25,37,44,55]. For example, discrimination or stigmatization from the social context can be significant triggering factors [42,44,55,57]. These adverse social dynamics can exacerbate the emotional challenges faced by individuals with T1DM [25,35,37,44,49,55,57].

#### 3.3.2. Nutrition

Nutrition plays a pivotal role in emerging adulthood, often marking a shift towards independence from parental dietary influence [43,44]. However, this period can be characterized by erratic eating patterns, often influenced by the demanding schedules of university or work, which can adversely affect postprandial glycemic stability [31,44,50,52]. Indicators of unhealthy food intake, as identified through self-reported information, include a high consumption of highly processed foods and refined grains, diets with high calorie density, predominantly carbohydrate-heavy meals, frequent fast food consumption, a low intake of vegetables, and overeating, particularly in settings such as buffets. Furthermore, a notable deficiency in the knowledge of carbohydrate counting persists in this age group, significantly compromising the accurate dosing of insulin [34].

In this context, healthcare providers recommend rigid and impersonal dietary plans, which often lead to a monotonous and unvaried diet [34,43]. This rigidity can be particularly challenging, given the critical importance of socialization in emerging adulthood, which frequently occurs through communal eating experiences [34,48,50,54]. The combination of limited knowledge in carbohydrate counting and the challenges associated with estimating carbohydrate content outside the home environment often results in dietary restrictions [34,48]. This may include attempts to avoid carbohydrate intake when eating out, thereby limiting the social and culinary experiences of individuals in this stage of life [43,52].

#### 3.3.3. Perceptions

In emerging adults with T1DM, personal perceptions play a critical role in interpreting and responding to their condition [26,52]. This research delves into the self-perception of individuals within this demographic category and how they perceive relevant aspects of their environment, inevitably influencing their self-view [26,38,52]. It is observed that such adults often consider themselves distinct from their peers, a distinction stemming from the unique challenges associated with managing their illness [24,27,29,41,48]. Additionally, a perceived lack of understanding is highlighted, where empathy is often found only among those sharing their condition [41,55].

The transition to adult or university life is identified as a significant challenge, compounded by the inherent demands of diabetes management [24,31,34,37]. A notable self-perception among some participants is a sense of invincibility, possibly inherited from adolescence, which can influence both their condition management and engagement in risky behaviors [27,33,34]. Socially, there is a discernible fear of rejection from peers and romantic partners, linked to the visibility of diabetes management devices or scars [27,54,57]. This situation is further complicated by the perception of dependence that arises from sharing blood glucose levels with peers or partners, leading to reluctance in disclosing information about glycemia levels [49,54,57].

Interestingly, many participants view diabetes as an integral component of their identity, leading to an increased perception of awareness, empathy, and maturity [24,26,36,37]. Some even perceive their condition as an opportunity to improve vital aspects of their life, such as lifestyle, quality of life, and mathematical skills, given the necessity for precise calculations in diabetes management [24,36,37,43].

These perceptions significantly impact the attitudes and behaviors of emerging adults with diabetes, profoundly affecting their lifestyle [44,47,49]. The acceptance of the disease, influenced by individual perception, leads to variations in the disclosure and normalization of self-care in daily life [25,26,34,35,37,43,44,45,50,56]. This phenomenon underscores the importance of a holistic understanding of these perceptions to facilitate the effective integration of diabetes management into the lives of emerging adults [23,33,35].

#### 3.3.4. Physical Activity

In the realm of physical activity, emerging adults with T1DM encounter various challenges, including social (college schedules, work pressures, or lack of time), environmental (lack of adequate space), and personal barriers, particularly in diabetes management during exercise [31,34,52]. These difficulties significantly impact their participation in regular physical activities [31,52]. Moreover, physical activity is often perceived as an imposed aspect of T1DM management, creating a complex relationship between the perceived benefits of exercise or the belief it is not necessary and the associated risks [35]. A key challenge for these individuals is achieving a balance between target glycemia and physical activity, considering how exercise influences blood glucose levels and necessitates adjustments in treatment [44,50].

The consequences of these dynamics are diverse. On one hand, a reduction in physical activity is observed due to the aforementioned barriers [50]. However, there are also instances of increased or maintained physical activity, motivated by the benefits of achieving target blood glucose levels [31,44]. This suggests that, despite the challenges, the recognized value of exercise in regulating blood glucose levels can encourage a continued commitment to physical activity among adults with T1DM [44,52].

#### 3.3.5. Risky Behavior

In the context of T1DM, emerging adults face significant challenges in integrating this condition into their adult roles, particularly in social activities such as attending events, parties, dances, and consuming alcohol [27,32,33,40,45,46].

A key issue is the fear of alcohol consumption due to a lack of knowledge about its impact on diabetes management and insulin regulation. It is important to note that this consumption of alcohol is neither approved nor guided by healthcare professionals, leading to a trend of normalization among these individuals, who often manage their alcohol intake through a trial-and-error approach [27,32,40,49]. This practice occurs despite being less frequent than among peers without T1DM. Simultaneously, the continuation of social activities without alcohol consumption is observed, reflecting a conscious effort by some individuals with T1DM to maintain an active social life while minimizing associated risks [49].

Another identified risky behavior is the omission of insulin and neglect of glycemic self-monitoring in social settings, often due to the challenges of managing these aspects of their condition in public or to avoid disclosing their T1DM [30,33,34,35,45,47,49].

Finally, the intentional maintenance of hyperglycemia is noted as a strategy adopted by some to cope with the uncertainties and challenges of diabetes management in social situations [30,33]. This behavior underscores the complexity of the decisions that emerging adults with T1DM must make to balance their health with their social life [45].

#### 3.3.6. Self-Care

In the context of self-care for emerging adults with T1DM, various factors have been identified that either facilitate or hinder this essential practice [55]. Key facilitators include the development of routines, schedules, and effective supply management, encompassing both physical and mental planning [25,36,37,41,47]. Crucial to this process is the interaction with friendly and communicative health professionals who understand and meet the realistic, individual needs of this life stage, moving beyond standardized care approaches [28,37,41,49]. Instrumental and emotional support from friends and family emerges as a crucial pillar in fostering appropriate self-care behaviors [34,35,36,37,50,51,52,54]. Furthermore, satisfaction with treatment and a willingness to integrate new technologies also significantly facilitate this process [24,25,56].

Conversely, barriers include interaction difficulties during the transition to adult care with punitive health professionals and a lack of specific, coherent information suitable for this vital stage [27,30,32,35,40]. Negative emotions and the underestimation risk, as described in the “Emotions and Feelings” section, irregular schedules, constant displacement, and the need to adapt to the intense, stereotypical rhythm of emerging adult life add to these challenges [27,32,34,35,41,48,54]. Other obstacles include the lack of social support, a limited space for storing supplies, the absence of competent T1DM professionals in university settings, inflexible clinical hours, bureaucratic processes, and extended waiting times [33,34,38].

A continual effort is observed to integrate self-care into daily life [24,29,31,44]. However, there is also a tendency to avoid medical appointments and even attempts to deceive health professionals [33]. A concerning behavior is the omission of glycemic goals, relying on self-perception of signs and symptoms, or ceasing insulin for special occasions, to the detriment of effective self-care [35,41,45]. Often, self-care is subordinated to organizational or group schedules, highlighting the complexity of managing T1DM amidst the demands of emerging adult life [24,31,32,35,47].

#### 3.3.7. Sleep

Sleep, a crucial aspect of health and well-being, presents both facilitators and barriers in emerging adults with T1DM. Among the facilitators are the implementation of routines, daytime physical activity, accumulated tiredness, a comfortable sleeping environment, and optimal glycemic goal at bedtime or setting an alarm for low glucose levels during the night [41,51,52]. The impact of sleep deprivation on diet is also noteworthy [51].

However, there are multiple barriers that affect sleep quality, including stress, anxiety, caffeine consumption, screen exposure, a lack of tiredness, variability in sleep schedules, worries, and having a full stomach [46,51]. Furthermore, social pressure to go to bed late and late bedtimes are seen as markers of the emerging adult lifestyle [50,51]. Additional disruptive factors include hypo/hyperglycemias, device alarms, and the need for nocturnal glycemic stability [51].

Optimal sleep reconciliation and duration are observed due to the greater weight of facilitators and less social pressure [51]. In contrast, sub-optimal sleep reconciliation and duration are attributed to the greater influence of barriers and increased social pressure. This can lead to increased food consumption and missed insulin doses due to social jet-lag [51].

#### 3.3.8. Social Relationships

In the context of social relationships for emerging adults with T1DM, a variety of factors significantly impact both their disease management and overall well-being [25]. Parents play a crucial role, engaging in a process of transferring responsibilities and providing instrumental, emotional, and financial support [26,30,35,39,42,45,47], although sometimes this support can result in becoming more burdensome for the emerging adult with T1DM [29,34].

Friends and peers often play an ambivalent role [26,43]. On one hand, they pressure the emerging adult with diabetes to conform to a stereotypical lifestyle, yet on the other hand, they provide significant emotional and instrumental support [27,30,32,33,35,46,47]. The support from partners is particularly noteworthy during episodes of hypoglycemia [53,54,57]. Disclosing diabetes to a partner has considerable physical and emotional impacts, but it is also accompanied by valuable emotional and instrumental support [53,54,57].

Similarly to peer relationships, interactions with healthcare providers vary; they sometimes are a source of support tailored to individual needs, but at other times, they can be counterproductive, where the emerging adult might conceal risky behaviors, such as alcohol consumption, to avoid judgment [25,29,31,34,35,36,37,39,40,41,43,44,46,48,50]. Work and academic environments, including relationships with coworkers or classmates, can be either a support, especially when they are diabetes-friendly and offer time and space for self-care, or an obstacle when lacking these conditions [25,28,32,34,37,51,53]. Peers with diabetes and diabetes camps are acknowledged as valuable sources of support [33,37,38,39,41,42]. From a spiritual and social perspective, religion and community associations provide emotional support and a sense of hope [37,38,42,57].

Maintaining supportive and trustworthy relationships is key in facilitating the social disclosure of the disease and seeking support, which in turn promotes proper self-care [45,55,56]. Conversely, an unfavorable environment can lead to concealing diabetes, worsening disease management [48,50,55,56].

#### 3.3.9. Stigma

In the context of T1DM, a range of misunderstandings and social judgments significantly impacts emerging adults with this condition [27,34,49,52]. Often, there is a social confusion that conflates states of drunkenness with hypoglycemia, leading to misinterpretations about the individual’s condition, which can even compromise their health [35,37,41]. Furthermore, insulin injections are sometimes mistakenly associated with drug addiction, and there is a prevalent confusion between T1DM and T2DM [25,37,40,44,48].

A particularly notable misconception is the perception of T1DM as a contagious disease [37]. This misunderstanding contributes to the stigma and challenges faced by individuals with T1DM in social settings [34]. Additionally, there is social judgment surrounding the consumption of food for treating hypoglycemia, often leading to unwarranted scrutiny and criticism [44].

Individuals with T1DM frequently face a dilemma: whether to disclose their condition to dispel these misconceptions, or to hide it to avoid judgment and stigmatization [25,27,35,41,54,55]. This choice reflects the broader challenge of managing a chronic illness that is poorly understood in a social context where misconceptions and judgments are common [27,34,48].

## 4. Discussion

This systematic review reveals the complex challenges involved in managing diabetes during emerging adulthood. It is the first of its kind to systematically review qualitative research and comprehensively understand the lifestyles of emerging adults with T1DM. While other reviews, such as the one by de Carvalho et al. [58], also focus on the global lifestyle aspects of individuals with T1DM, they are based on quantitative studies and cover a range from adolescence to emerging adulthood. Although the review by Monaghan et al. [9] on emerging adulthood with diabetes and lifestyle considerations is a significant contribution, it does not describe systematic methods in the synthesis of results or assessments of data reliability.

This review highlights that emerging adulthood often leads to a mix of emotions and significant stress as individuals endeavor to balance effective blood glucose management with the social, academic, and occupational demands characteristic of this life stage [46]. According to the qualitative findings from the research by Kelly et al. [59], older individuals with T1DM experience less distress compared to younger ones, and generally, more challenges arise externally, especially among the younger population. Similarly, in our findings, emerging adults with diabetes often compare their experiences with those of their peers without T1DM. They particularly notice differences in aspects such as choosing healthy foods or the necessity to count carbohydrates before meals [24,34,37,43,50].

These emotional and social challenges underscore the necessity of effective management strategies, as highlighted in the review by Monaghan et al. [9]. The synthesis of the reviewed studies underscores the importance of planning a timetable and adhering to a meticulous daily regimen. This method is identified as a key strategy for reducing perceived differences compared to peers without T1DM and for managing potential unwanted glycemic variability [25,35,36,39,52,56]. Another aspect emphasized, in both the direct quotes from the selected studies and the results of the meta-aggregation, is the need for anticipation. This relates to blood glucose levels and insulin administration, as well as the preparation of necessary supplies. Such a requirement for foresight affects the spontaneity usually associated with this stage of life [24,25,35,36,39,48,52,56]. Considering all these factors, it can be posited that, at times, the establishment of regular schedules and routines might dictate the lifestyle of emerging adults with T1DM [54,56].

In addition to these management strategies, the literature reveals that academic and work schedules, particularly shift work, pose significant disruptions, leading to a complex interplay between self-care routines and the pursuit of autonomy among emerging adults with T1DM. Over half of the studies analyzed concluded that academic and work schedules, particularly shift work, disrupt self-care routines, leading to feelings of frustration and stress for emerging adults with T1DM as they strive to synchronize their self-care practices with institutional timetables [24,25,27,28,29,30,31,33,34,35,36,38,39,44,46,50,51,52,54,56]. In this context, time management and utilization are perceived as burdensome. Additionally, although parents continue to play a role in care routines, there is an increasing need for greater self-care autonomy among emerging adults, reflecting their desire for more independence [26,56]. Similarly, various studies included in this review [27,28,30,32,33,34,37,47,50] reveal that the perception of this burden intensifies when the information on T1DM management provided by healthcare providers or educational settings is limited, unclear, or restrictive. This observation becomes especially evident in relation to typical behaviors for this age group, such as alcohol consumption [32].

The systematic analysis of qualitative evidence provides insight into how emerging adults with T1DM exhibit various behaviors influenced by their self-perception and the degree to which they accept their diabetes [34,43]. For instance, the presence of negative emotions and perceiving diabetes as a burden are known to hinder the integration of self-care, particularly in social settings [29,45,49,52]. This often leads to behaviors such as intentionally neglecting diabetes management in social contexts, including the deliberate omission of insulin dosing, glycemic monitoring, or carbohydrate counting [29,45,49,52]. Such behaviors may also arise from stressful life events, as highlighted by Joiner et al. [60]. On the other hand, when positive emotions allow individuals to view diabetes as an opportunity for self-care, and when the acceptance and normalization of the condition are integrated in daily life, managing care becomes more feasible [9]. This perspective fosters behaviors like engaging in physical exercise, maintaining proper nutritional management, or seeking social support, without avoiding diabetes self-care in public settings [42,43,55].

The integration and acceptance of T1DM are crucial for developing lifestyle habits such as physical activity practice. Although the study by Anjali et al. [61] highlights facilitators and barriers of exercise for emerging adults, both the scoping review by Brennan et al. [62] and the qualitative study by Vlcek et al. [63] focus specifically on the exercise barriers and facilitators for individuals with T1DM, spanning life from childhood to old age. This focus is particularly relevant given the comparative lack of attention to the impact of inactivity in individuals with T1DM, especially when contrasted with the attention given to those with T2DM [64,65]. The scarcity of time and the tight schedules of emerging adults with T1DM make them particularly vulnerable to the harms of a sedentary lifestyle [64]. Furthermore, the meta-analysis by Huerta-Uribe et al. [66] reveals that from childhood, those with T1DM are significantly more inactive and sedentary than their counterparts without T1DM, suggesting the strong impact of specific barriers [63].

Concerning this, the reviewed body of literature has facilitated the compilation of various barriers and facilitators that shed light on the management of T1DM in social contexts [9]. Key facilitators include strong social support, especially from friends and institutions, and increased self-awareness [26,38,42]. Conversely, the literature identifies several barriers, such as stigma [29,48], a tendency to downplay the severity of the disease [47], and a desire to be perceived as equal in social interactions [32,41]. However, no concrete barrier has been demonstrated through quantitative methodology [67]. In the realm of romantic relationships, while the quantitative study by Pinhas-Hamiel et al. [68] does not identify concerns related to sex beyond hypoglycemia, this systematic review reveals specific challenges stemming from fears of rejection due to the use of body devices like continuous glucose monitoring (CGM) and its impact on self-image [57]. Additionally, there is a need to establish boundaries in self-care to promote independence and prevent overbearing dynamics [57]. Thus, this systematic review exposes a consistent dilemma in deciding whether to conceal or disclose T1DM management, influenced by its potential positive or negative effects on social interactions.

Thus, based on the synthesis conducted in this review, it can be inferred that most emerging adults with T1DM typically endeavor to integrate behaviors common to their age group into their daily lives. This intricate balancing act extends beyond mere scheduling; most emerging adults with T1DM are also endeavoring to integrate age-typical behaviors, such as socializing and partaking in leisure activities, into their diabetes management routines [52]. Such behaviors include alcohol consumption, smoking, staying up late on weekends, or engaging in spontaneous outings [24,25,27,34,35,37,39,45,50]. At times, the avoidance of these behaviors results from a lack of understanding about how to incorporate them into their self-care routine without causing glycemic imbalance [24,30,33,37,40,49,56]. This observation is in line with the findings of Valerio et al. [69] in their quantitative study. While to a lesser extent compared to their peers without T1DM, the study reveals that tobacco and alcohol consumption are also present among adolescents with T1DM. Furthermore, there is a notable lack of qualitative research on sleep, even though partial results about self-perceptions and living with diabetes have been found in this review. Except for one article, findings specifically related to sleep are scarce, despite evidence from Larcher et al. [70] that social jet lag worsens glycemic stability. Nevertheless, anticipation, planning, and trial-and-error are underscored as essential strategies to navigate these situations or to balance academic and work responsibilities with diabetes self-care [24,25,35,36,39,52,56].

A notable strength of this review is its focus on the first-hand experiences of emerging adults with T1DM, a demographic less frequently studied compared to others such as children or adolescents. Furthermore, this review’s methodological rigor is enhanced by the inclusion criterion that necessitates high-quality research, as determined by the JBI Critical Appraisal Checklist for Qualitative Research. This criterion has significantly contributed to the review’s overall quality and validity, aligning with the standards suggested by Creswell and Miller [71]. In addition, another key strength of our review is the implementation of the JBI’s CONQual approach, ensuring a rigorous assessment of the confidence in qualitative findings [23]. This methodology allowed for a nuanced evaluation of the findings’ dependability and credibility, contributing significantly to the robustness of our conclusions [23].

Every study encompassed in this review utilized a qualitative methodology, typically involving fewer participants compared to quantitative studies. The optimal sample size for qualitative research is guided by the concept of data saturation [72], leading to a notable range in sample sizes, from as few as seven (N = 7) to as many as sixty-seven (N = 67). While the sample size in qualitative studies is not inherently a limitation, it is noteworthy that 7 out of the 10 reviewed articles [25,27,30,35,41,42,43]—those involving 10 or fewer participants—do not explicitly address the saturation point. This omission presents a notable gap in the current evidence, warranting consideration in the interpretation of these studies and this systematic review.

In systematic qualitative reviews, the diversity of study settings can be seen as a strength, potentially enhancing the transferability of the findings [73]. However, the variation in countries and cultures necessitates a cautious interpretation of results [74]. It is important to acknowledge that health services and their offerings differ significantly across countries [30]. Such variations can notably influence the impact of health education on nutrition, especially regarding recommended diets, whether they are based on planned menus or carbohydrate counting. Additionally, the nature of a health system, be it public or private, along with access to health resources and the affordability of medical devices like continuous subcutaneous insulin infusion pumps, can affect aspects of self-care, social relationships, and perceptions of stigma. While these differences may not be considered strict limitations, they are essential for understanding, interpreting, and applying this review’s findings [30].

Innovative technologies, such as CGM systems and automatic insulin infusion systems, are pivotal in managing T1DM among emerging adults [75,76]. These tools assist in monitoring glycemic levels, recognizing patterns in physical activities and dietary habits, and preventing glycemic imbalances [76]. They are particularly effective in enhancing self-care by providing real-time data and facilitating better sleep management through features like alarm programming and insulin delivery suspension, thus contributing to overall better diabetes management and decision making in self-care [77]. However, it is important to acknowledge the potential stigma from the visible use of these devices, which can lead to feelings of self-consciousness. Healthcare providers should respect individuals’ decisions regarding the adoption of these technologies, ensuring that the benefits are communicated while understanding and addressing any concerns about stigma. This approach fosters a supportive environment, empowering individuals in their diabetes management choices.

Moreover, the legal and social definitions of adulthood vary by region, which can influence the results. Lastly, it is worth noting that behavioral particularities are discussed in the reviewed body of literature with minimal mention of lifestyle factors, with the exception of one study [54], which does incorporate ‘lifestyle’ as a key term.

The primary limitation of our study arises from the non-inclusion of the term ‘emerging adult’ in our search strategy, as it is absent from the DeCS and MeSH thesauri. This absence presented a challenge in specifically targeting results related to emerging adults with T1DM. Consequently, we employed the descriptors ‘Young Adult’ (encompassing adults up to 24 years old) and ‘Adult’ (including those up to 44 years old). This approach inevitably broadened the age range beyond our initial inclusion criteria, resulting in the exclusion of a substantial number of studies whose samples did not conform to our specified age limits. In addition to these primary search terms, we conducted a thorough examination of secondary literature and performed a citation verification using Google Scholar, adhering to the PRISMA guidelines.

Although qualitative studies and their corresponding reviews do not permit the generalization of results, they are crucial for comprehending the knowledge, attitudes, and lifestyles of emerging adults with T1DM [78,79]. This review, by focusing on the behaviors that shape the lifestyles of emerging adults with T1DM, offers a holistic framework for health professionals to use [80]. Proper clinical follow-up, aimed at promoting healthy lifestyle, may enhance their quality of life. This is particularly significant for conditions like T1DM, where improving the quality of life is a key therapeutic goal due to the lack of a cure [81]. Based on our findings, we recommend further research into diverse coping strategies and improvements in communication and education by healthcare providers to address identified barriers. Additionally, the development of specific interventions to tackle these barriers and enhance facilitators identified in each category is suggested. As a future research avenue, it is imperative to emphasize the importance of longitudinal qualitative and quantitative studies to assess the long-term effects of lifestyle changes and their health outcomes.

In developing control programs for emerging adults with T1DM, identifying key behavioral factors is vital for shaping effective interventions. Essential factors include the practice of regular self-monitoring of glucose levels and adjusting insulin based on objective data rather than physical sensations. Proficiency in carbohydrate counting and appropriate insulin dosing in response to dietary intake is also critical. Planning and anticipatory management for scenarios like exercise or alcohol consumption are necessary to maintain glycemic balance. Additionally, fostering a positive attitude towards diabetes and empowering patients to handle social stigma are crucial for enhancing self-management. Focused interventions on these behavioral aspects—self-monitoring, carbohydrate counting, anticipatory management, and positive attitude—can significantly empower emerging adults in managing T1DM effectively.

Additionally, such research aligns with Goal Three of the United Nations’ 2030 Agenda for Sustainable Development, which focuses on promoting health and well-being at all ages. By addressing the specific needs of emerging adults with T1DM, this research contributes to the broader objective of enhancing global health, underlining the significance of this often-neglected age group in public health policies and initiatives [82].

## 5. Conclusions

This systematic review has underscored the importance of qualitative research in deepening our understanding of the unique lifestyle challenges and behavioral motivations faced by emerging adults with T1DM. Our findings reveal a complex interplay between diabetes management and the pursuit of autonomy, highlighting the need for tailored interventions that address specific lifestyle aspects, including diet, sleep, substance use, and sexual health.

Key behavioral factors such as self-monitoring, carbohydrate counting, anticipatory management, and a positive attitude towards diabetes management emerged as crucial in facilitating effective self-care among this demographic. Moreover, this review identifies a significant gap in research on the lived experiences of emerging adults with T1DM in areas like sleep, risky behaviors, and sexual relationships, suggesting the need for further qualitative and quantitative studies in these less explored domains.

The insights gained from this review point towards the need of developing health promotion strategies that are specifically designed for emerging adults with T1DM. Such strategies should not only focus on clinical aspects of diabetes management but also consider the broader lifestyle and psychosocial factors that influence self-care behaviors. It is imperative that future research continues to explore these areas, contributing to more comprehensive care approaches that support the overall well-being and quality of life for individuals with T1DM during this critical stage of life.

## Figures and Tables

**Table 1:** Study characteristics.

	Year	Study Location	Authorship	Research Techniques	Sample Total (Women) (Age Range)	Categories Addressed
1	2010	Sweden	Sparud-Lundin, C. et al. [26]	SI and IC	N = 13(7); (20–22)	EF. P. SR.: (family, peers, and peers with T1DM).
2	2011	United States	Pyatak, E. [27]	SI	N = 8(4); (19–25)	EF. P. RB. SC. ST and SR: (health care provider).
3	2012	United Kingdom	Snow, R. and Fulop, N. [28]	SI	N = 17(9); (18–25)	EF. SC. and SR.: (work and environmental, social support: health care provider). ST.
4	2013	Ireland	Balfe, M. et al. [29]	SI	N = 35(29); (23–30)	P. SC. SR.: (work, family, and health care provider). ST.
5	2013	United States	Pyatak, E. et al. [30]	SI	N = 8(5); (19–25)	EF. RB. SC. SR.: (social support: family, friends, and health care provider).
6	2014	Ireland	Balfe, M. et al. [31]	SI	N = 35(29); (23–30)	EF. N. P. PA. SC. SR.: (environmental and work colleagues).
7	2014	United States	Garvey, K.C. et al. [32]	FG	N = 26(16); (25–27)	EF. SC. RB. SR: (health care provider, peers with T1DM).
8	2014	United States	Pyatak, E. et al. [33]	SI	N = 20(11); (20–22)	EF. P. RB. SC. SR.: (environmental, college, and health care provider).
9	2015	Turkey	Celik, S. et al. [34]	I	N = 28(16); (18–25)	EF. N. P. PA. RB. SC. ST. SR.: (family, peers, and health care provider).
10	2015	United States	Hood, D.G. and Duke, G. [35]	SI	N = 9(6); (19–24)	EF. P. RB. SC. ST. SR.: (environmental, family, peers, and health care provider).
11	2015	Canada	Visekruna, S. et al. [24]	SI	N = 9(9); (22–30)	EF. P. SC. SR.: (environmental and health care provider).
12	2016	United States	Fredette, J. et al. [36]	SI	N = 24(21); (18–24)	EF. P. SC. SR.: (environmental, peers, peers with T1DM, family, and health care provider).
13	2017	United States	Abdoli, S. et al. [37]	I	N = 9(6); (18–30)	EF. P. SC. ST. SR.: (environmental, peers, peers with T1DM, family, health care provider, and God).
14	2017	Canada	Clausi, L. and Schneider, M. [25]	SI	N = 7(7); (18–22)	EF. P. SC. ST. SR: (peers).
15	2017	United States	Fedor, A. et al. [38]	SI	N = 13(9); (18–22)	SC. SR.: (environmental, peers, peers with T1DM, and health care provider).
16	2017	United States	Pierce, J.S. et al. [39]	SI	N = 10(7); (18–25)	P. SC. SR.: (environmental, family, peers, peer with T1DM, and health care provider).
17	2018	United States	Calamaro, C.J. et al. [40]	QS	N = 12(7); (19–26)	EF. RB. SC. ST. SR.: (peers with T1DM and health care provider).
18	2018	Sweden	Hilli, Y. et al. [41]	SI	N = 8(4); (20–25)	EF. P. SC. SL. ST. SR.: (peers with and without diabetes, associations, family, and health care provider).
19	2018	South Africa	Visagie, E. et al. [42]	SI	N = 8(5); (18–25)	EF. SR.: (family, peers, peers with T1DM, and health care provider).
20	2018	South Africa	Willemse, M. et al. [43]	I(LS)	N = 8(3); (20–25)	EF. N. P. PA. SR.: (family, friends, and health provider)
21	2019	United States	Addala, A. et al. [44]	FG	N = 17(11); (18–25)	EF. N. P. PA. SC. ST. SR.: (health care provider, work, partner, and environmental).
22	2019	Sweden	Carlsund, Å. and Söderberg, S. [45]	SI	N = 12(8); (19–30)	EF. P. RB. SC. SR.: (family, peers, and health care provider).
23	2019	United States	Ersig, L. A. [46]	QS	N = 25(2); (18–24)	EF. RB. SL. SR.: (family and peers).
24	2019	Canada	Markowitz, B. et al. [47]	I	N = 33(17); (18–24)	EF. P. RB. SC. SR.: (family, peers, and health care provider).
25	2019	Australia	Mullan, B. A. et al. [48]	SI	N = 25(21); (20–30)	EF. N. P. SC. ST.
26	2019	United States	Ramchandani, N. et al. [49]	FG	N = 21(15); (18–29)	EF. P. RB. SC. ST. SR.: (family, peers, and health care provider).
27	2019	United States	Saylor, J. et al. [50]	FG	N = 12(5); (19–26)	EF. N. P. PA. RB. SL. SR.: (family, peers, and professor at university).
28	2020	United States	Griggs, S. et al. [51]	SI	N = 30(20); (19–25)	EF. SL.
29	2020	United States	Hanna, K. M. and Hansen J. R. [52]	SI	N = 25(12); (19–26)	N. P. PA. SC. SL. ST. SR.: (family, peers, workmates or supervisors, and others).
30	2020	Portugal	Santos, A. N. et al. [53]	QS	N = 59(47); (18–30)	SC. SR.: (partner).
31	2021	United States	Gray, A. L. et al. [54]	SI	N = 29(16); (22–24)	N. P. SC. ST. SR.: (family, peers, and partner).
32	2021	Canada	Habenicht A. E. et al. [55]	SI	N = 14(7); (18–28)	EF. P. SC. ST. SR.: (family, peers, partners, and work/classmates).
33	2021	United States	Skedgell, K. K. et al. [56]	SI	N = 62(29); (18–24)	P. SC. SR: (family, peers, peers with T1DM, and health care provider).
34	2021	United States	Yorgason, J. B. et al. [57]	FG	N = 12(7); (22–30)	EF. P. SR.: (partner).

Abbreviations: Research techniques: Focus Group (FG); In-depth Interviews (I); Internet Chat (IC); Life Story (LS); Qualitative Survey (QS); Semi-structured Interviews (SI). Categories addressed: Emotions and Feelings (EF); Nutrition (N); Perceptions (P); Physical Activity (PA); Risky Behavior (RB); Self-Care (SC); Sleep (SL); Social Relationships (SR); and Stigma (ST).

## Data Availability

The data presented in this study are available upon reasonable request. The data are available from María-Ángeles Núñez-Baila (email: mnbaila@us.es).

## Supplementary Materials

The following supporting information can be downloaded at: https://www.mdpi.com/article/10.3390/healthcare12030309/s1, Table S1: Step-by-step process of the meta-aggregation of qualitative findings. Table S2: Confidence in the findings: Application of CONQual.
