# Supplementary material for: Lifestyle in Emerging Adults with Type 1 Diabetes Mellitus: A Qualitative Systematic Review
Source: Healthcare (Basel). 2024 Jan 25;12(3):309. doi: 10.3390/healthcare12030309 (PMC10855310; doi:10.3390/healthcare12030309)
Supplement: Supplementary file 1 [file healthcare-12-00309-s001.zip › Supplementary Material Table S1- Meta-Aggregation_Revised.pdf]

**Supplementary Material Table S1 – Meta-Aggregation**

Table S1. Step-by-Step Process of Meta-Aggregation of Qualitative Findings.

| Article | Key Findings Identification | Grouping of Similar Findings | Findings Synthesis | Category based on PI(E)CO |
|---|---|---|---|---|
| 1 | **Evolution of the parent-child relationship** during the transition to adulthood.<br>Parental and significant others' support strategies.<br>**Challenges in self-identity** and integrating diabetes into this identity. | **Transformation in supportive relationship**s and their impact on diabetes management.<br><br>Reflective considerations and adjustments in **perceptions of self and diabetes.** | Transition to adulthood for those people managing diabetes involves significant changes in family support and self-care responsibilities. This phase sees a shift from parental guidance to greater self-management, promoting individual autonomy.<br>Individuals adapt their self-identity, integrating the condition into their self-understanding. Influenced by changing relationships and social dynamics, this adaptation leads to an ongoing reassessment of how they choose to disclose or conceal their condition in social contexts | Emotions and Feelings, Perceptions, Social Relationships: family, peers and peers with T1DM. |
| 2 | **Emotional responses** to diabetes, including distress, feelings of loss of balance, and resentment.<br>**Challenges in negotiating unanticipated events** due to a perceived sense of invincibility.<br>**Embodied knowledge of diabetes**, including awareness of bodily signs and tolerance limits.<br>**Access to and satisfaction with healthcare,** influencing treatment adherence.<br>**Impact of shifting physical contexts** on diabetes self-management.<br>**Role of social support**, sensitivity to others' | **Emotional and psychological aspects:** emotional responses, negotiation of events, and social perceptions.<br><br>**Practical and environmental factors:** embodied knowledge, healthcare interactions, physical contexts, and routines. | A complex interplay of emotional, psychological, and practical challenges faced by emerging adults living with T1DM. Emotional distress and societal perceptions significantly impact their diabetes self-management practices. These challenges, coupled with the complexities of navigating healthcare systems and daily routines, underscore the heightened risk of both acute and long-term diabetes-related complications. The effectiveness of self-management is influenced by each individual's physical environment, healthcare experiences, and ability to integrate diabetes care into daily life, all of which are crucial in mitigating these risks. | Emotions and Feelings, Perceptions, Risk behaviour, Self-Care, Stigma and Social Relationships: environmental, family, peers, and healthcare providers. |

| | | | | |
|---|---|---|---|---|
| | perceptions, and experiences of stigma.<br>**The influence of schedules**, routines, and special events on diabetes management. | | | |
| 3 | **Complex patterns of clinic attendance**, including non-attendance and cancellations.<br>**Administrative and communication challenges** leading to false 'missed' appointments.<br>**Patients' cost-benefit analysis** of attending clinics versus obstacles faced.<br>**Importance of practical information**, emotional support, and reassurance in clinic visits.<br>**Influence of clinic hours on accessibility** for patients with unsympathetic employers.<br>**Negative experiences** with health professionals affecting future clinic attendance.<br>**Role of routine and parental support** in consistent attendance, and phases of 'denial' in disease management. | **Systemic and administrative barriers:** communication issues, clinic hours, and administrative procedures.<br><br>**Personal and emotional factors:** perceived value of clinic visits, experiences with healthcare staff, and emotional responses to the condition. | The multifaceted challenges faced by individuals in managing diabetes clinic appointments. Systemic barriers such as inefficient communication and rigid clinic hours compound personal struggles, including dealing with unsympathetic employers and negative interactions with healthcare professionals. These factors collectively influence patients' decisions regarding clinic attendance, often leading to a complex pattern of engagement with healthcare services. Emotional support and practical information are highly valued by patients, but negative experiences can significantly deter future attendance, indicating a need for more patient-centric and flexible healthcare approaches. | Emotion and Feeling, Self-Care and Social Relationships: healthcare provider. |
| 4 | **Differing attitudes towards weight** between male and female interviewees, with females showing more concern.<br>Female interviewees' **concerns about weight** gain due to insulin and difficulties in losing weight.<br>Instances of insulin omission for weight balance, discovered accidentally and perceived initially as a positive experience.<br>**Escalation of disordered eating behaviors** | **Psychological and behavioral aspects:** Concerns about weight, insulin omission for weight loss, and the development of eating disorders.<br>**Systemic and familial influences**: challenges in healthcare diagnosis and treatment, and the role of family in either supporting or exacerbating eating disorders. | A complex interplay between diabetes management, body image, and eating disorders among young adults. Females expressed significant concerns about weight gain linked to insulin use, leading to dangerous practices like insulin omission. This behavior often started inadvertently but escalated into more severe eating disorders, profoundly impacting their health and daily life. The findings also highlight systemic shortcomings in healthcare and the varied role of family support, pointing to a need for more comprehensive and sensitive | Perceptions. Self-Care, Social Relationships: work colleagues, family, and healthcare provider. |

| | | | | |
|---|---|---|---|---|
| | and their eventual negative impact on daily life and health.<br>**Role of family support** in the context of eating disorders, with varying degrees of effectiveness.<br>**Difficulties in the healthcare system** to diagnose and treat young women with eating disorders effectively. | | approaches to address these intertwined issues. | |
| 5 | **Variability in adherence to diabetes management,** with some participants following recommendations and others consistently making nonadherent decisions.<br>**Influential factors for adherence** include collaborative healthcare approach, supportive family and friends, and satisfaction with treatment.<br>**Nonadherence influenced by factors** like misleading healthcare providers, adherence to alternative standards, treatment fatigue, social support issues, and emotional and self-efficacy challenges.<br>**Diverse experiences with healthcare providers** and geographic mobility impacting care continuity. | **Psychological and behavioral aspects:** efforts to mislead healthcare providers, emotional and self-efficacy issues, and treatment fatigue.<br>**Systemic and environmental influences:** The role of healthcare provider approach, family dynamics, and geographic mobility in treatment adherence. | The study uncovers a complex array of factors influencing diabetes management adherence among young adults. A collaborative approach by healthcare providers, along with supportive social networks, significantly boosts engagement. Conversely, non-engagement is often driven by psychological factors such as treatment fatigue and self-efficacy issues, as well as systemic challenges like varying healthcare experiences and lack of consistent support. These findings emphasize the need for personalized and empathetic healthcare strategies that address the diverse needs and challenges faced by individuals with diabetes. | Emotions and Feelings, Risk Behavior, Self-Care and Social Relationships: family, peers, and healthcare providers. |
| 6 | **The workplace environment** characterized by long working hours and high intensity, impacting diabetes management.<br>**Difficulty in integrating diabetes management** into the workday, often leading to neglect of management tasks during busy periods. | **Work-related factors:** time pressures, non-routine environments, and work stress impacting diabetes management.<br>**Personal and psychological factors:** Attitudes towards diabetes care in the context of work and life pressures. | Significant challenges emerging adults with diabetes face in managing their condition within demanding and non-routine work environments. Time pressures, workplace stress, and the need to conform to workplace norms often lead to compromised diabetes management. These findings highlight the necessity for flexible and adaptable diabetes care strategies that can be integrated | Emotion and Feelings, Nutrition, Perceptions, Physical Activity, Self-Care, Social Relationships: |

| | | | | |
|---|---|---|---|---|
| | **Pressure to conform to workplace norms**, leading to minimized diabetes management activities.<br>**Challenges in maintaining routine diabetes care** due to non-routine work environments and additional responsibilities.<br>**Impact of workplace stress** on eating habits and blood sugar levels.<br>**Difficulties in managing diabetes due to travel and lack of structured routines in unemployment**. | | into diverse and dynamic work schedules, as well as the importance of supportive workplace policies and practices. | environmental and work colleagues. |
| 7 | Participants experienced an unintentional and **unprepared transition** to adult diabetes care, often having to find a new provider independently.<br>**Vulnerability during college years** with reduced clinical follow-up and challenges in diabetes self-care.<br>**Unexpected differences between pediatric and adult care systems,** with a focus on older patient populations and more prevalent type 2 diabetes in adult care.<br>Emerging adults expressed a **desire for improvements in the transition process**, including more structured counseling and targeted referrals to adult diabetes providers. | **Transition process: lack of planned transition and need for more structure and support** in shifting to adult care.<br>**Experiences during college year**s: Specific challenges related to university life and managing diabetes during this period (risk behaviors: sexuality and alcohol consumption).<br>**Systemic differences:** Contrast between pediatric and adult care, and emotional impact of facing chronic diabetes complications.<br>**Recommendations for improvement:** Patient suggestions for facilitating the transition and enhancing the care experience. | Emerging adults with T1DM often face an unstructured and challenging transition from pediatric to adult care. This transition is characterized by a lack of preparation and guidance, leading to vulnerabilities during college years and surprise at the differences in adult care. Participants express a desire for a more intentional and supported transition, with specific guidance and peer support to adapt to the new reality of adult diabetes care. | Emotion and Feelings, Self-Care, Risk behavior, Social Relationships: healthcare providers, peers with T1DM. |
| 8 | **Health provider/System challenges:** Emerging adults reported minimal guidance on transitioning to adult care, with a lack of clear information and appropriate referrals | **Transition guidance and support:** Consistent lack of structured transition planning and support from pediatric to adult care. | A critical gap in structured transition support for emerging adults with T1DM. The participants' experiences highlight the complexities of navigating the healthcare system, along with developmental and | Emotions and Feelings, Perceptions, Risk behaviors, Self- |

| | | | | |
|---|---|---|---|---|
| | from pediatric providers. Misunderstandings about healthcare financing and navigating public healthcare systems were common.<br>**Developmental challenges:** Emerging adults faced disruptions in care due to feelings of invincibility, poor planning, and deprioritizing medical needs amidst competing priorities.<br>**Psychosocial challenges:** Participants' care was often hampered by urgent demands stemming from their life circumstances, including family issues, safety concerns, and lack of knowledge in navigating the health system. | **Understanding and navigating healthcare:** difficulties in understanding healthcare options, eligibility, and dealing with bureaucratic challenges in the healthcare system.<br>**Developmental and psychosocial factors:** influence of typical young adult behaviors and complex life situations on diabetes management. | psychosocial factors (sense of invincibility) that complicate diabetes management during this transition phase. These findings emphasize the need for targeted interventions (risk behaviors) and enhanced communication strategies to facilitate a smoother transition from pediatric to adult healthcare for emerging adults with T1DM. | Care, Social Relationships: Environmental – college and healthcare providers. |
| 9 | **Negative emotions about the disease:** Emerging adults experience a range of negative emotions like fear, frustration, and shame, leading to tendencies to hide the disease and non-compliance with insulin and diet.<br>**Difficulties arising from living conditions:** Challenges in professional and educational environments impact diabetes management, with conflicts between managing the disease and social expectations.<br>**Treatment process challenges:** The attitude of the treatment team, especially judgmental and non-empathetic behaviors, affects the patients' willingness to follow-up and adhere to recommendations. | **Emotional and psychological challenges:** The complex emotional and psychological landscape of emerging adults, including fear, frustration, and coping mechanisms.<br>**Social and environmental constraints:** The influence of social interactions, workplace or educational settings, and societal perceptions on diabetes management.<br>**Healthcare system and provider interaction:** The impact of treatment team's approach and healthcare system navigation on disease management. | Multifaceted challenges faced by emerging adults with T1DM, encompassing emotional, social, environmental, and healthcare-related factors. These challenges often interplay, creating a complex scenario that hampers effective diabetes management. Emotional struggles, combined with social pressures and inadequate support systems, lead to poor adherence to treatment and lifestyle modifications. The findings call for a more integrated approach in diabetes care, addressing not only the medical aspects but also the emotional, social, and psychological needs of emerging adults. | Emotion and Feelings, Nutrition, Physical Activity, Perceptions, Self-Care, Risk behavior, Stigma and Social Relationships: family, peers, and healthcare providers. |

| | | | | |
|---|---|---|---|---|
| | **Lack of social support:** Issues with trust in families and friends, peer pressure, and societal prejudices contribute to the challenges in diabetes management.<br>**Non-solution oriented Coping methods:** Feelings of weakness, avoidance of problems, and blaming others are prevalent.<br>**Concerns about the future:** Fears about long-term complications and effects on future life aspects, including relationships and family.<br>**Developing knowledge and attitude regarding diabetes management:** Understanding the necessity of insulin, blood sugar checks, diet, and exercise but facing challenges in incorporating these into daily life | | | |
| 10 | **Seeking stability:** Emerging adults experienced a significant struggle for managing their diabetes management, especially when transitioning to insulin pumps and adjusting to new life environments like university.<br>**Becoming responsible:** The journey towards independence in diabetes management was marked by key life events like getting a driver's license and facing real-life complications.<br>**Staying connected:** The need for a support network was crucial, highlighting feelings of isolation and the importance of connection | **Emotional and psychological adaptation:** Challenges in managing diabetes, especially the emotional journey in accepting the disease and the responsibility that comes with it.<br>**Social dynamics and support system:** The importance of support systems, including the transition from pediatric to adult healthcare and the impact of feeling connected or disconnected in managing diabetes.<br>**Self-management and independence:** Transitioning to self-management involves not only understanding and | The challenges of maintaining blood glucose levels in range, coupled with the psychological and social aspects of living with diabetes, underscore the need for comprehensive support systems. Accepting one's condition and integrating it into their identity is a crucial part of this journey. These insights suggest a need for personalized care strategies that address not only the medical aspects but also the emotional, social, and practical challenges faced by emerging adults with T1DM. | Emotion and Feelings, Perceptions, Self-Care, Stigma, Risk Behaviour and Social Relationships: environmental, family, peers, and healthcare providers. |

| | | | | |
|---|---|---|---|---|
| | with healthcare professionals, family, and peers. **Accepting me:** Emerging adults with T1DM underwent a personal journey of accepting their condition, transitioning from hiding their diabetes and insulin pumps to embracing them as part of their identity. | using technology like insulin pumps but also coping with the physical, practical, and social implications of diabetes care. | | |
| 11 | **Elusiveness of management:** Participants emphasize the challenge of maintaining stable blood glucose levels, with routine and consistency being key yet often difficult to achieve. The fear of complications and the impact on future plans, such as family planning, are recurrent concerns. **Forecasting and maintaining routines:** The importance of routines in diabetes management is highlighted, though adapting these routines to fit individual lifestyles and dealing with unpredictable schedules, especially in university or work settings, poses challenges. **Dualism of technology:** Insulin pump therapy offers flexibility and stability, but also presents challenges such as the difficulty of concealing the device and trusting its suggestions. Continuous glucose monitoring (CGM) is seen as a valuable tool, though its use varies based on personal preferences and life stages. **Dealing with 'Ups and Downs':** Participants grapple with the physical and psychological | **Challenges in achieving blood glucose target:** The continuous struggle for stable blood glucose levels amidst varying life circumstances. **The role of technology in diabetes management:** Balancing the benefits and challenges of insulin pumps and CGM in achieving better glycaemic stability. **Psychosocial and emotional aspects:** The impact of diabetes on mental well-being, lifestyle adjustments, and relationships with healthcare providers. **Striving for balance:** The pursuit of equilibrium in managing diabetes, encompassing self-care, routine, and adapting to life changes. | The complex nature of managing T1DM among young women, highlighting the elusive nature of maintaining glycaemic stability and the significant role of technology in facilitating management. It underscores the need for adaptable routines and strategies to deal with the unpredictable nature of diabetes, particularly in relation to lifestyle changes, exercise, and menstrual cycles. Emotional and psychological challenges are prevalent, stemming from the fluctuating nature of the disease, weight management issues, and interactions with healthcare providers. Ultimately, these experiences converge into the central theme of seeking balance, underscoring the need for holistic approaches in diabetes care that consider both the physical and emotional aspects of the condition. | Emotion and Feelings, Perceptions, Self-Care and Social Relationships: environmental and healthcare providers. |

| | | | | |
|---|---|---|---|---|
| | impacts of fluctuating blood sugar levels, including the effects of exercise, menstrual cycles, and stress. Managing weight alongside diabetes is also a noted struggle.<br>**Interfacing with the healthcare team:** Experiences with healthcare teams vary, with some participants preferring the more personalized approach in pediatric care compared to adult care. The relationship with healthcare providers is crucial, yet often marked by frustration, particularly in managing expectations and receiving adequate support.<br>**The essence of 'Being in Balance':** The overarching goal is achieving a balance in life with diabetes, incorporating self-management strategies, adapting to life's unpredictability, and aligning personal goals with diabetes management. | | | |
| 12 | **Planning ahead:** Participants transitioning to college and independent living had to carefully plan daily activities and diabetes management. This included pre-exam blood glucose monitoring, adjusting to a new routine, and coping with the challenges of managing blood glucose during physical activities, social events, and academic responsibilities.<br>**Thinking positive:** Many participants reported a positive impact of T1DM on their lives. They felt it contributed to increased | **Adapting to new challenges:** Planning ahead and seeking support are strategies to adapt to the new challenges of managing T1DM in a college environment.<br>**Psychosocial development:** Developing a positive mind-set and utilizing support systems contribute significantly to psychosocial growth and effective diabetes management. | The complexities of managing T1DM during the transition to college life. Effective management requires meticulous planning and adaptation to new routines. The participants' ability to maintain a positive outlook, despite the challenges, illustrates resilience and an initiative-taking approach to health. The role of support systems, both formal (diabetes organizations) and informal (family and friends), is pivotal in providing the necessary guidance, emotional support, and sense of community. These findings underscore the need for tailored support strategies to assist emerging adults with T1DM in successfully navigating this critical life | Emotion and Feelings, Perceptions, Self-Care, Social Relationships: environmental, peers, peers with T1DM, family, and healthcare providers. |

| | | | | |
|---|---|---|---|---|
| | responsibility, maturity, and health awareness. Despite the challenges, they were able to find optimism and personal growth through their condition.<br>**Seeking support:** support from peers, family, diabetes organizations, and online communities was crucial. This support helped them in coping with T1DM management during college, offered a sense of belonging, and provided a platform for sharing experiences and advice. | | transition. | |
| 13 | **Struggle toward adulthood:** Emerging Appalachian adults with T1DM navigate the complexities of growing up, balancing the transition to adult responsibilities with diabetes management.<br>**Daily struggles of living with T1DM:** These include financial challenges, insurance limitations, healthcare access, management difficulties, community unawareness, employment issues, dating challenges, and social stigma.<br>**Supportive environment:** Despite struggles, a strong support system from family, friends, healthcare providers, and workplace helps emerging adults to cope.<br>**Strong desire to live life:** Participants exhibit a resilience to live life fully, despite T1DM limitations, focusing on making diabetes management enjoyable and connecting with peers. | **Adulthood transition and identity formation:** Combining struggles toward adulthood and identity development reflects how T1DM influences personal growth and self-perception.<br>**Daily management and societal challenges:** Grouping struggles of living with T1DM, social stigma, and technological challenges underscores the multifaceted daily life complexities faced by these individuals.<br>**Support and positive outlook:** The themes of supportive environment and strong desire to live life highlight the importance of external support and internal resilience. | The multifaceted experiences of emerging Appalachian adults with T1DM. It underscores the intricate balance between the challenges of managing T1DM and the transition into adulthood. The findings reveal how T1DM significantly shapes personal identity and daily life, posing both physical and psychosocial challenges. Despite these difficulties, there is a notable resilience and positivity among participants, supported by strong familial, social, and healthcare networks. The role of technology emerges as a double-edged sword, offering enhanced management tools yet creating new dependencies. Overall, these insights underscore the need for holistic support systems that address both the medical and psychosocial aspects of living with T1DM in emerging adulthood. | Emotion and Feelings, Perceptions, Self-Care, Stigma, Social Relationships: environmental, peers, peers with T1DM, family, healthcare providers, and God. |

| | | | | |
|---|---|---|---|---|
| | **Development of identity:** T1DM shapes their identity positively, leading to a healthier lifestyle, better numeracy, and advocacy for diabetes awareness.<br>**Disruptiveness of diabetes' demands:** The permanence of T1DM brings daily fears and concerns about long-term complications and life-threatening situations.<br>**Pros and cons of technological development:** While technology aids in diabetes management, it also introduces new challenges and dependencies. | | | |
| 14 | **Desire for freedom:** Young women with T1DM expressed a desire for more freedom from the constant self-management routines, especially regarding insulin pump use. They felt restricted and self-conscious due to the visibility of their pumps.<br>**Integration of diabetes into identity:** Emerging adults viewed diabetes management as a regular part of their routine, with many considering it integral to their identity, having adapted to living with it from an emerging adulthood.<br>**Striving for normalcy:** The women expressed a wish to be perceived as normal, without their diabetes defining them. They felt different from their peers and frustrated by the lack of control over their condition. | **Psychological impact of diabetes management:** The constant need for management, visibility of insulin pumps, and the unpredictability of blood glucose levels led to feelings of frustration, self-consciousness, and a sense of being different.<br>**Identity and self-perception:** Diabetes management had become a normalized part of their identity, yet they struggled with the desire to be seen as normal and not solely defined by their diabetes. | The complex interplay between the practical and psychological aspects of managing T1DM in emerging adult women. The desire for freedom and normalcy conflicts with the ever-present nature of diabetes management. Despite integrating diabetes into their identity, these women grapple with the visibility of their condition and its impact on self-perception. The findings underscore the need for support systems that address not only the physical but also the emotional and social challenges of living with T1DM. | Emotion and Feelings, Perceptions, Self-Care, Stigma, Social Relationships: peers. |
| 15 | **Transition in medical care:** Most emerging adults with T1DM continued care with their | **Healthcare and transition challenges:** Continuity in medical care but logistical | A key aspect is the continuity of medical care from pediatric to adult providers, accompanied by logistical | Self-Care, Social Relationships: |

| | | | | |
|---|---|---|---|---|
| | original endocrinologists after transitioning to college. Challenges in scheduling appointments due to academic commitments and travel difficulties. No gaps in medical care reported during the college transition phase. Positive experiences with registered dietitians (RDs) and certified diabetes educators (CDEs), despite some feeling that HCPs lack personal diabetes experience.<br>**Resources:** basic campus resources include student health services, counseling, a recreation center, and dietitian services. Mixed experiences with campus health services, particularly in diabetes management knowledge. Suggestions for campus improvements include having an on-campus diabetes specialist or CDE.<br>**Friendship and Support:** friends, including those with diabetes, cited as a crucial support system.<br>Participants naturally gravitating towards peers with diabetes for mutual understanding and support. | challenges in maintaining regular appointments. Positive role of specialized diabetes healthcare professionals.<br>**Campus resource limitations and suggestions:** limited diabetes-specific support and knowledge on campus. Recommendations for enhanced campus resources, including specialized diabetes care professionals.<br>**Social support networks:**<br>significant role of friendships in diabetes management.<br>Shared experiences and empathy among peers with diabetes. | challenges in scheduling and traveling for appointments. The positive impact of specialized diabetes healthcare professionals' contrast with the general lack of diabetes-specific knowledge and resources on college campuses. Students suggest the need for more tailored resources, such as on-campus diabetes specialists.<br>Social support emerges as a vital element, with friendships providing emotional and practical support. Particularly, connections with peers who also have diabetes offer a unique understanding and empathy. This social network is instrumental in helping students navigate the complexities of diabetes management within the dynamic college environment. | environmental, peers, peer with T1DM, and healthcare providers. |
| 16 | **Biomedical markers of T1DM management:** emphasis on maintaining or decreasing HbA1C levels, with concerns about over-reliance on HbA1C as a sole indicator.<br>**Navigation of a new healthcare system:** Importance of establishing continuity in | **Medical and self-management aspects:**<br>• **Biomedical markers and self-management skills**: Both focus on the individual's ability to manage their T1DM effectively through knowledge and medical indicators. | Successful healthcare transition for emerging adults T1DM is marked by a multifaceted integration of autonomy and proficiency in disease management. Those who navigate this transition adeptly maintain biomedical levels, as evidenced by appropriate HbA1C levels, and exhibit effective self-management skills. Navigating the adult healthcare system involves | Perceptions, Self-Care. Social Relationships: environmental, family, peers, peers with T1DM, and |

| | | | | |
|---|---|---|---|---|
| | healthcare post-transition and forming collaborative relationships with adult T1DM specialists.<br>**Possession of T1DM self-management skills and knowledge:** Necessity for knowledge and skills for daily self-management, including executive functioning skills.<br>**Integration of T1DM care into emerging adult roles:** Learning to prioritize T1DM self-management amidst adult responsibilities and social relationships.<br>**Balance of parental involvement with autonomy:** Finding a balance between parental support and the emerging adult's independence in managing T1DM.<br>**Attainment of T1DM "Ownership":** emphasis on emerging adults' acceptance of their condition and proactive engagement in T1DM management. | • **Integration of T1DM care and ownership:** These themes revolve around the individual's integration of their T1DM management into their daily lives and taking personal responsibility.<br>**Healthcare system and support networks:**<br>• **Navigation of new healthcare system and parental involvement:** These findings relate to external support systems, including healthcare providers and family, essential for successful transition and management. | establishing collaborative relationships with healthcare providers and striking a balance between parental involvement and independent self-care. A unifying element across these aspects is the attainment of 'ownership' of the disease, where the individual acknowledges T1DM as an integral part of their identity and actively assumes responsibility for their care. In summary, a successful transition is multidimensional, blending medical, personal, and supportive elements to foster independent and effective diabetes management in adulthood. | healthcare providers. |
| 17 | **Lack of specific health information:** T1DM resources have been predominantly catered to children and adolescents, often sidelining emerging adults. This gap was highlighted in participants' experiences, where educational materials and healthcare communication were more child-centric, leaving emerging adults feeling under-informed and overlooked.<br>**Emotional turbulence and forward thinking:** The diagnosis of T1DM in | **Communication needs/concerns:** The diagnosis process and subsequent interactions with healthcare providers play a crucial role in setting the stage for self-management and adherence. Emerging adults expressed a desire for more straightforward, empathetic communication and a need for information delivery that acknowledges their autonomy for develop.<br>**Physical needs/concerns**: Managing | There is a significant gap in terms of information and interventions designed for emerging adults with type 1 diabetes. This is due to the healthcare system traditionally focusing on childhood and adolescence for type 1 diabetes, and on type 2 diabetes during adulthood. There are psychological and physical challenges in this population, which reports experiencing stigmatization from society and calls for interventions tailored to lifestyle changes and the adoption of stereotypical behaviours such as alcohol consumption, as well as the support of mental healthcare | Emotion and Feelings, Risk Behaviour, Self-Care, Stigma and Social Relationships: peers with diabetes and healthcare providers. |

| | | | | |
|---|---|---|---|---|
| | emerging adults precipitates a range of emotional responses, from shock and grief to acceptance and forward-thinking. This emotional journey highlights the need for psychological support and education in managing both immediate and long-term aspects of the condition.<br>**Specific needs and concerns of young adults with T1DM:** This theme encompasses communication, physical, and psychosocial needs unique to this age group. Communication with healthcare providers emerged as a critical area, with an emphasis on the need for clear, direct, and empathetic dialogue.<br>• **Physical concerns** focused around managing diabetes in a college environment and maintaining a lifestyle consistent with their peers without T1DM.<br>• **Psychosocial needs** included the desire for peer support, stigma management, and the need for counseling services focusing on young adults with chronic conditions. | T1DM in a college setting presents unique challenges, including access to medical supplies and specified education in diabetes to sort out typical lifestyles like alcohol consumption. Concerns about the impact of T1DM on academic pursuits and lifestyle were also prominent.<br>**Psychosocial needs/concerns:** Emerging adults with T1DM face psychosocial challenges, such as dealing with stigma and the emotional impact of living with a chronic condition. The need for peer support and access to mental health services was strongly emphasized. | and social support. | |
| 18 | **Factors promoting motivation for self-care:**<br>• **Feeling safe and receiving support:** Emerging adults value practical support, such as reminders for self-care activities. Living a structured | **Promoting self-care motivation:**<br>• Feeling Safe and Supported: emphasizes the fluctuating nature of self-care motivation, dependent on support systems | Self-care motivation in emerging adults with T1DM is multifaceted, influenced by both internal and external factors. The presence of a supportive environment, management of one's life, and a sense of wellbeing significantly bolster self-care motivation. In contrast, | Emotion and Feelings, Perceptions, Self-Care, Sleep, Stigma. Social |

| | | | | |
|---|---|---|---|---|
| | life facilitates the integration of self-care into daily routines and improves self-care, as well as a higher level of acceptance of the disease.<br>• **Experiencing management of one's life:** for emerging adults with T1DM possessing the necessary skills for proper self-care is crucial. Additionally, when a diabetes nurse considers their life situation and preferences, it empowers them to feel they manage their own lives and simultaneously enhances their motivation for self-care.<br>• **Striving for wellbeing and meaning:** a positive attitude and engaging in work, physical exercise, and leisure activities provide exceptional motivation for self-care. Wellbeing is both a goal and a motivational factor, but it should be assessed in the short term through metrics like blood glucose levels, rather than long-term indicators like HbA1c.<br>**Factors that hindered motivation for self-care**<br>• **Feeling vulnerable:** vulnerability arises when the demands of diabetes self-care clash with stressful social | and personal maturity.<br>• Management and autonomy: highlights the importance of a structured life and the use of aids for managing T1DM effectively.<br>• Wellbeing and meaning: focuses on physical and psychological wellbeing, and its role in motivating self-care.<br>**Hindering Self-Care Motivation:**<br>• Vulnerability: describes how stress and societal pressures negatively impact self-care.<br>• Exclusion: details how feelings of alienation and misunderstanding by others can hinder motivation for self-care. | feelings of vulnerability and exclusion, often due to societal misconceptions and pressures, pose substantial barriers to consistent self-care. These findings suggest a need for a holistic approach to emerging adults with T1DM | Relationships: peers with and without diabetes, associations, family, and healthcare providers. |

| | | | | |
|---|---|---|---|---|
| | obligations faced by the emerging adult. This can lead to denial of the illness and an acceptance of potential future complications.<br>• **Feeling excluded:** stigma in society due to low literacy can lead to the concealment of self-care practices in social contexts. This fosters a sense of exclusion in public settings and a reduction in self-care. | | | |
| 19 | **Social support agents:** This encompasses family, friends, and healthcare providers. Family members provide companionship and emotional support; healthcare providers offer informational support and guidance.<br>**Mechanisms of social support actions:** These include ongoing support from family, healthcare providers, and peers. Support manifests as practical, financial, and emotional assistance, as well as diabetes education and awareness. | **Familial and emotional support:** encompasses emotional, financial and practical support from family members, such as accompanying to medical appointments, dietary adjustments, and assistance in diabetes management.<br>**Healthcare professional support:** focuses on diabetes knowledge and management, providing essential information and practical support.<br>**Peer and friend support:** includes practical support in day-to-day diabetes management and emotional support, such as being aware of specific needs and assisting in social situations. | Effective diabetes management in emerging adults hinges on a composite support system encompassing family, friends, and healthcare professionals. Familial support -primarily emotional, practical, and financial- is vital in fostering companionship and managing daily challenges. Healthcare providers offer crucial informational support, enhancing understanding and management of diabetes. Friends augment this support by providing both practical assistance and emotional solidarity, demonstrating the importance of a well-informed social network. The collaboration of family, peers, and healthcare professionals creates a dynamic support framework, addressing the diverse needs of diabetes management. This research highlights how awareness and education within one's social circle contribute to effective diabetes care and emotional well-being, reinforcing the idea that a multi-dimensional support network is essential for navigating the complexities of diabetes management in emerging adults. | Emotion and Feelings, Social Relationships: family, peers, peers with T1DM, and healthcare providers. |
| 20 | **Identity development:** Challenges in | **Combating identity challenges:** | A new identity that incorporates diabetes management | Emotion and |

| | | | | |
|---|---|---|---|---|
| | identity development are pronounced among emerging adults with T1DM, as they strive to balance normalcy with their condition.<br>**Personal management and subjective well-being:** Managing diabetes involves a journey of personal empowerment and subjective well-being, despite the challenges of adhering to strict diets, exercise schedule and lifestyle modifications.<br>**Social support:** Family and friends provide crucial support, assisting in practical management and offering emotional encouragement.<br>**Health-care counselling support:** Medical professionals, including endocrinologists and counselors, play a vital role in providing education, guidance, and emotional support.<br>**Futuristic goals**: Despite the initial setbacks in career and personal aspirations due to diabetes, participants express determination to overcome these challenges and manage their condition. | Emerging adults are navigating the complexities of establishing their identities while integrating diabetes management into their lives.<br>**Empowerment through self-management:** There is a strong focus on personal empowerment, learning to listen to their bodies, and making informed management decisions.<br>**Integral role of social networks:** The support from family, friends, and healthcare professionals is indispensable in providing both practical help and emotional reassurance.<br>**Adapting to new realities:** Participants express a need to adjust their aspirations and goals considering their diabetes yet remain optimistic about overcoming these challenges. | as an integral part. This process involves a significant degree of personal empowerment, as individuals learn to make informed decisions about their health and lifestyle. The role of social support, encompassing family, friends, and healthcare providers, emerges as a cornerstone in their life, offering necessary practical and emotional sustenance. Moreover, the findings reflect the participants' resilience in adapting their life goals and aspirations to accommodate their condition. | Feelings, Nutrition, Perceptions, Physical Exercise, and Social Relationships: family, friends, and health provider. |
| 21 | **Dual burden of T1DM and weight management:** Emerging adults describe the frustrating interplay between managing T1DM and weight, noting that it turns their lives into a series of numerical evaluations.<br>**Hypoglycemia as a barrier to weight balance:** Treatment of hypoglycemia is seen as a major obstacle to effective weight | **Challenges in dual management:** Combining the management of T1DM and weight presents unique challenges, exacerbated by societal stigma and hypoglycemia.<br>**Technology and information needs:** There is a clear desire for more information and support regarding | Emerging adults with T1DM face complex challenges in managing both their condition and weight. They navigate hypoglycemia as a barrier to weight balance, confront societal stigmas, and express a need for personalized diabetes technology. Transitioning to adulthood introduces new dimensions: romantic relationships, financial burdens, and work commitments significantly impact T1DM and weight management. | Emotion and Feelings, Nutrition, Perceptions Physical Activity, Stigma, Self-Care, Social Relationships: |

| | | | |
|---|---|---|---|
| management, particularly due to the need for carbohydrate intake and the impact on exercise routines.<br>**Stigma surrounding T1DM and weight:** Emerging adults perceive, and experience stigma related to both T1DM and weight, stemming from misinformation and societal pressures, particularly affecting women.<br>**Desire for greater technology integration:** There is a notable interest in more detailed information and personalized guidance regarding T1DM-specific technology, including insulin pumps and phone-based apps.<br>**Needs in research studies:** Emerging adults express a desire for a multidisciplinary, individualized approach in research studies, emphasizing the need for peer support groups and consistent communication.<br>**Role of insulin in weight management:** Participants report a complex relationship between insulin intake and weight management, noting the detrimental effects of insulin purging.<br>**Prioritization of glycemic stability over weight loss:** Glycaemic target is prioritized over weight loss, with a focus on exercise as a means to achieve optimal health.<br>**Unhealthy eating patterns:** Eating patterns are influenced by work demands, leading to fasting and binging behaviours. | diabetes-specific technology and a call for individualized guidance.<br>**Interpersonal relationships and external influences:** The role of family, partners, and societal expectations significantly impacts the management of T1DM and weight.<br>**Healthcare dynamics:** Evolving relationships with healthcare providers and the need for more supportive and understanding medical care are crucial themes. | In emerging adulthood, specific challenges emerge. Concerns about family planning and fertility, alongside evolving relationships with medical providers, highlight the unique struggles in this life stage. These findings underscore the need for nuanced support as emerging adults balance T1DM management with the intricacies of adult life. | family, peers, and healthcare providers. |

| | | | | |
|---|---|---|---|---|
| | **Personal relationship with T1DM:** Participants reflect on their internalization of comments about T1DM and the importance of self-advocacy in managing their condition.<br>**Incorporating responsibilities of adulthood:** Themes of romantic partnerships, financial considerations, work obligations and family planning are discussed in relation to T1DM and weight management.<br>**Role of medical providers:** There is a growing tension in the relationship with medical providers, with participants reporting non-supportive approaches and a desire for a more empathetic and helpful engagement. | | | |
| 22 | **Handling the situation:**<br>• **Managing daily life:** Emerging adults, from an early age, demonstrated a resolute approach towards managing their long-term illness independently, including self-administration of insulin and blood glucose monitoring. A majority expressed a need for taking their own decisions in planning daily activities, reflecting a deep-seated sense of responsibility for their illness from a young age. Notably, participants appreciated | **Self-management and personal control:** A strong emphasis on self-management and personal control over their condition was evident, with participants taking an active role in their daily diabetes care.<br>**Emotional and mental health impacts:** Managing diabetes is not only a physical challenge but also an emotional and mental one, with stress, mood swings, and feelings of frustration being common.<br>**Social dynamics and understanding:** The interaction with others revealed a spectrum of understanding about | Emerging adulthood is marked by an intense sense of autonomy and a desire of managing their condition, counterbalanced by the emotional toll of constant vigilance. The supportive role of healthcare professionals is highly valued, providing a contrast to the challenges faced in social settings, particularly in managing the impacts of alcohol on their condition. The social aspect of living with diabetes is multifaceted, characterized by a mixture of support and misunderstanding from others. A recurrent theme is the struggle with sustaining motivation for continuous self-care, reflecting the ongoing challenge of balancing diabetes management with the desire for a normal life. | Emotion and Feelings, Perceptions, Risk behaviour. Self-Care, Social Relationships family, peers, and health provider. |

the positive attitudes and support from healthcare professionals, but also faced challenges like navigating alcohol consumption and its impact on diabetes management

- **Emotional roller coaster:** The management of diabetes was often described as stressful, with constant worries about blood glucose levels being too high or too low. Participants shared fears of losing control during hypoglycemic episodes and described significant mood swings associated with fluctuating glucose levels.

**Dealing with different opinions:**

- **General attitudes and concerns:** Participants encountered various responses from others, ranging from curiosity and interest to misunderstandings and lack of knowledge about T1DM. They expressed frustration with misconceptions and highlighted the support they received from family and friends.
- **Own views and apprehensions:** Participants generally didn't feel ashamed of their condition and received strong support from family and friends. However, some chose diabetes, from supportive to lacking. The importance of social support and the challenges of dealing with misconceptions about the condition were evident.

**Challenges in sustaining motivation:** The fluctuation in motivation levels, from diligent self-care to periods of neglect, underscores the ongoing challenges in living with T1DM

| | | | | |
|---|---|---|---|---|
| | to hide their illness in certain situations due to negative comments and misunderstandings. Despite overall support, feelings of loneliness and a desire for more understanding were expressed. There was a common frustration with the public's misconceptions about the illness. Participants appreciated the empathetic and humanizing approach of healthcare professionals.<br>• **Ignorance and lack of motivation:** all participants at some point experienced a lack of motivation in managing their illness, with some avoiding insulin injections or struggling to maintain consistent management. This was attributed to the challenges of living with a chronic condition and the desire for normalcy. | | | |
| 23 | **Life stage stress**: Stress is centered around typical college life challenges such as academic pressures, social relationships, and future career anxieties.<br>**Diabetes management worries:** Students face ongoing concerns with daily diabetes management, struggling with blood sugar control amidst their busy college schedules, sleep, and involving in risk behaviour like | **Stress and anxiety across life stages:** Stress focusing on immediate college life challenges.<br>**Chronic disease management in a dynamic environment:** Emerging adults struggle to maintain diabetes control in the fluctuating schedule of college life.<br>**Future uncertainties:** Concerns about future implications of T1DM, including | The multifaceted challenges faced by emerging adults with T1DM with managing their condition amidst the pressures, schedules, and lifestyles of college life. The interplay of immediate diabetes management challenges with longer-term worries about health and life implications is a persistent theme. Additionally, the critical role of social networks in emergency situations underscores the importance of community awareness and support in managing chronic conditions like T1DM. | Emotion and Feelings, Risk behaviour, Sleep, and Social Relationships: family and peers. |

| | | | | |
|---|---|---|---|---|
| | alcohol consumption. Emerging adults lived with uncertainty inherent blood sugar management and control.<br>**Long-term implications and complications:** Emerging adults with T1DM are concerned about the long-term implications of T1DM, including health insurance coverage, future relationships, and potential diabetes complications.<br>**Importance of college-based social network:** The reliance on social networks for emergency diabetes management becomes vital, with students educating their peers about T1DM. | personal relationships and health complications are prevalent.<br>**Social support systems:** The crucial role of social networks in managing diabetes emergencies highlights the importance of community support in chronic disease management. | | |
| 24 | **Ingrained lens:** describe a seamless integration of diabetes into daily life, viewing it as a comfortable and manageable aspect of their identity. Emerging adults adopted a practical and flexible approach management. Moreover, the help of parents and support of healthcare providers are welcome.<br>**Intrusive lens:** characterizes diabetes as a burdensome and constant worry, significantly impacting daily life management. Emerging adults with T1DM felt overwhelmed, distressed, and experiencing a lack of motivation.<br>**Inconspicuous lens:** involves minimizing the impact of diabetes, often avoiding management tasks to preserve a sense of | **Diabetes management approaches:** Different approaches to diabetes self-management are evident, ranging from effective integration into daily life to perceptions of diabetes as an intrusive or minimized aspect of life. | There is a spectrum of experiences among emerging adults with T1DM, characterized by varied narrative lenses. The ingrained lens indicates a successful integration of diabetes into one's life, whereas the intrusive lens reflects ongoing struggles and burdens. The inconspicuous lens suggests a tendency to minimize the impact of diabetes, possibly as a coping mechanism. | Emotion and Feelings, Perceptions. Risk behaviour. Self-Care and Social Relationships: family, peers, and healthcare providers. |

| | | | | |
|---|---|---|---|---|
| | normalcy in social settings. In contrast to intrusive lens, these emerging adults portrayed themselves as not especially burdened. | | | |
| 25 | **'Simple' becomes complicated:** Participants highlighted the complexity of managing daily activities due to T1DM, expressing frustration with the additional calculations and considerations needed for routine tasks like eating and exercising.<br>**Constant vigilance:** A theme of continuous vigilance and monitoring was prevalent, with participants expressing fatigue, frustration, and sometimes burnout from the relentless nature of T1DM management.<br>**Controlling the 'uncontrollable':** Emerging adults used metaphors to describe the unpredictable nature of T1DM, indicating that despite their best efforts, managing the condition often felt like an art form filled with uncertainty and risks.<br>**Private becomes public:** Management of T1DM often led to uncomfortable situations in public, as participants navigated the reactions of others to their self-management activities, like injecting insulin or testing blood glucose levels.<br>**Myths of the 'diabetic':** Participants discussed the misconceptions and stereotypes associated with diabetes, particularly the confusion between T1DM | **Challenges in daily management:** The complexities of T1DM turn simple daily tasks into complicated processes, requiring continuous attention and adjustment. It leads to emotional strain, including feelings of burnout and frustration and sometimes the sensation of uncontrollable management.<br>**Social perception and stigma:** Managing T1DM in public settings often leads to misunderstandings and uncomfortable interactions. Thereby, emerging adults with T1DM face misconceptions and judgment about their management of diabetes, particularly around the distinctions between T1DM and T2DM or aspects of their lifestyles, which affects how they are perceived and treated by others. | Daily life activities are complicated by the need for constant management and vigilance, impacting both physical and emotional well-being. Participants experience a tension between managing their condition privately and the public exposure of their self-care practices, often leading to uncomfortable social interactions and public scrutiny. Misconceptions about diabetes, especially the confusion between T1DM and T2DM, contribute to a stigma that affects how individuals with T1DM are perceived and treated. This misunderstanding extends to health moralism, where participants face judgment about their lifestyle choices that are governed by the necessities of managing T1DM. | Emotion and Feelings, Nutrition, Perceptions, Self-Care, and Stigma. |

| | | | | |
|---|---|---|---|---|
| | and T2DM the stigma attached to the latter. **Health moralism:** Participants experienced judgment or unsolicited advice from others about their lifestyle choices and health management, reflecting a lack of public understanding about the intricacies of living with T1DM. | | | |
| 26 | **Finding a balance between diabetes and life:** Participants struggle with integrating diabetes management into the new independence and transitions of emerging adulthood, including college, work, and social life. **The desire to have a good management of their diabetes:** Despite challenges, participants strive for optimal glycemic stability, acknowledging that better control leads to feeling better, but also express frustration with the constant attention diabetes requires. Desire to feel normal, privacy or secrecy about their diabetes, fear of hypoglycemia and just giving up for a moment are the reasons for diabetes mismanaging. **The hidden burden of diabetes:** The ever-present nature of diabetes management, often unnoticed by others, is a significant challenge, with participants feeling the need to keep their condition private or worrying about public reactions. They mark drinking | **Balancing diabetes management:** The demands of emerging adulthood, such as academic stress and socializing, is a key challenge. The pursuit of control over diabetes is a common goal, yet it is accompanied by the frustration of managing a complex and demanding condition. The hidden burden of diabetes involves managing the condition discreetly and dealing with social anxiety or fear of public reactions. **Provider relationships:** a strong, supportive relationship with diabetes providers is crucial, with a need for providers to understand and address the broader life context of patients. | The transition to greater independence brings new complexities in managing diabetes, where finding a balance between daily life and diabetes care is a constant struggle. Participants want to manage their condition, yet this stability is often hindered by the relentless demands of diabetes management. The hidden burden of diabetes is a significant theme, where managing the condition is an internal, often private struggle that is not always apparent to others. This leads to challenges in social situations and a desire to maintain normalcy without drawing attention to their condition. The relationship with diabetes providers is pivotal. Participants desire a partnership approach, where providers are not just addressing medical needs but also understanding the life challenges and situation of each individual. This is a holistic approach to effective diabetes management and overall well-being. | Emotion and Feelings, Perceptions, Risk behaviours, Self-Care, Stigma and Social Relationships: family, peers and health provider. |

| | | | | |
|---|---|---|---|---|
| | alcohol also a hidden burden.<br>**The desire to have a connection with their diabetes provider:** participants express a strong desire for a supportive and communicative relationship with their diabetes provider, emphasizing the importance of being seen as a whole person in middle of transition phase, not just a patient with diabetes. | | | |
| 27 | **Diabetes affects all aspects of life and complicates college living:** Participants discuss how a T1DM diagnosis influences their college experience, impacting decisions like college choice and complicating daily college activities, including socializing and managing academic responsibilities. The spheres which the glycemic target is vulnerable are: diet, exercise, stress and sleep.<br>**College environment affects diabetes management:** The unique aspects of college life, such as dining hall options, class schedules, and campus layout, present specific challenges for managing T1DM.<br>**Diabetes diagnosis facilitates growth and maturity:** Participants reflect on how their T1DM diagnosis has accelerated their emotional growth and maturity, changing their outlook on life and responsibilities.<br>**Strategies used for diabetes management** | **T1DM and college life:** balancing T1DM management with the typical challenges of college life, such as academic stress, social activities, and independence, is a key theme. The college environment, including aspects like dining facilities and class planning, directly influences diabetes management.<br><br>**Personal growth and adaptive strategies:** The diagnosis of T1DM has led to increased responsibility and a more mature perspective on life and health. Therefore, emerging adults use various strategies to manage T1DM, including seeking social and institutional support, learning through experience, and advocating for their needs. | T1DM affects all life aspects of emerging adults, from academics to social interactions, requiring a constant balancing act. The unique college environment poses specific challenges for diabetes management, influencing food choices, physical activity, sleep, and overall routine.<br><br>The experience of living with T1DM in college is not only a challenge but also an opportunity for growth. Emerging adults report accelerated maturity, increased responsibility, and a changed outlook on life. They navigate their condition using a variety of strategies, from seeking support networks to actively learning through trial and error. Self-advocacy emerges as a crucial skill, especially in negotiating the college environment and managing health. The college should be a tailored support system for students with T1DM. It highlights the importance of understanding the unique challenges faced in the college setting and the need for strategies that support both diabetes management and personal development. | Emotion and Feelings, Nutrition, Perceptions, Physical Activity, Risk behaviour, Sleep and Social Relationships: family, peers, professor at university. |

| | | | | |
|---|---|---|---|---|
| | **in college:** different strategies are: Seeking support, accepting diagnosis, glycemic stability through trial and error, managing daily activities, and self-advocacy. | | | |
| 28 | **Barriers to obtaining sufficient sleep:**<br>• **General barriers:** common barriers included poor sleep hygiene, stress, and uncomfortable sleep environments. Factors like screen time, caffeine use, and irregular sleep schedules were also noted.<br>• **Diabetes-specific bedtime delays or disruptions:** Diabetes management, such as dealing with hyperglycemia or hypoglycemia and managing equipment like sensors and pumps, frequently disrupted sleep. Fear of hypoglycemia also emerged as a significant concern affecting sleep patterns.<br>**Facilitators to obtaining sufficient sleep:**<br>• **General facilitators:** Strategies like a regular relaxing bedtime routine, physical activity, a comfortable sleep environment, and avoiding caffeine were common. Using distractions like electronics or reading to relax before sleep was also mentioned.<br>• **Diabetes-specific facilitators:** keeping blood glucose within range before bedtime was crucial. This | **Sleep challenges:** General and diabetes-specific barriers, highlighting how poor sleep hygiene, stress, and diabetes management intertwine to impact sleep quality. It encapsulates the multifaceted nature of sleep disturbances, encompassing both lifestyle factors and the complexities of diabetes care.<br>**Strategies for sleep enhancement:** general and diabetes-specific facilitators for obtaining sufficient sleep. It underscores the significance of establishing relaxing bedtime routines and effective diabetes management before sleep. The role of both behavioral adaptations and conducive environmental conditions in promoting restorative sleep for emerging adults with diabetes. | Poor sleep hygiene, stress, and an uncomfortable sleep environment are general barriers to obtaining sufficient sleep. In the context of diabetes, managing blood glucose levels and equipment at bedtime poses additional challenges, with the fear of hypoglycemia being a significant concern.<br>To facilitate better sleep, emerging adults adopt various strategies. These include establishing a regular bedtime routine, engaging in physical activities during the day, creating a comfortable sleep environment, and using relaxation techniques such as meditation or light exercise. For diabetes-specific facilitators, maintaining blood glucose within a desired range before bedtime is critical. This involves careful meal planning, snack management, and effective use of diabetes equipment. | Emotion and Feelings, and Sleep. |

| | | | | |
|---|---|---|---|---|
| | included strategies like having a snack or light meal, avoiding eating close to bedtime, and managing diabetes equipment effectively. | | | |
| 29 | **Habits, triggers, and routines:** Emerging adults identified routine diabetes management activities, such as checking blood glucose and administering insulin, triggered by specific cues like timing, location, and internal state. Meals and exercise were also described as part of their regular routines. **Indicators of transitional disruption:** Transitional disruptions, including forgetting and disorder, were identified. These disruptions impacted habits and routines around meals, exercise, and overall diabetes management, particularly during transitions such as moving to college. **Influential factors:** Several factors influencing diabetes management were noted, including the conduciveness of the environment, stress, diabetes-related stigma, social support, and self-reliance. These factors variously supported or hindered effective diabetes care. | **Routine management and environmental triggers:** combines the aspects of routine diabetes management activities and the environmental and internal triggers that initiate these routines.<br><br>**Impact of life transitions on diabetes management and influential factors:** life transitions, like moving to college, disrupt established diabetes management routines and habits. Various external (environmental conduciveness, social support, stigma) and internal (stress, self-reliance) factors that influence diabetes management during these life transitions. | Routine diabetes care activities are often prompted by specific cues and are integral to effective self-management. However, life transitions, such as moving to college, can disrupt these established routines, leading to challenges in maintaining consistent diabetes care. The conduciveness of the environment, level of social support, and presence of diabetes-related stigma can either facilitate or hinder effective diabetes care. Internal factors such as stress and self-reliance also significantly impact how individuals manage their condition.<br><br>. | Nutrition, Perceptions, Physical Activity, Self-Care, Sleep, Stigma, and Social Relationships: family, peers, workmates or supervisors and others. |
| 30 | **Relationship adjustment:** Emerging adults with T1DM navigate their romantic relationships, emphasizing the importance of communication, self-disclosure, and mutual understanding of the illness within | **Interpersonal dynamics in relationships:** combines aspects of relationship adjustment and sexual and reproductive health. The theme described experiences focusing on how | Relationship dynamics are significantly influenced by T1DM, requiring of open communication and a deep understanding of the disease within the relationship. This mutual comprehension is vital for navigating the challenges and adjustments that T1DM brings to | Self-Care, Social Relationships: partner. |

| | | | | |
|---|---|---|---|---|
| | the relationship context. There is a bidirectional influence between romantic relationships and T1DM management.<br>**Knowledge needs:** informational needs of emerging adults with T1DM related to sexual and reproductive health. it includes the need for practical information about managing T1DM in relation to sexual activity and understanding how the disease interacts with aspects of sexual health, such as libido and childbirth planning.<br>**Sexual and reproductive health experience:** this theme reflects the emotional, cognitive, and physical experiences of emerging adults with T1DM in relation to their sexual and reproductive health. It covers their expectations, concerns, and the strategies they have developed to manage both T1DM and their sexual and reproductive health. | T1DM affects romantic relationships and sexual health, and vice versa.<br>**Informational and educational needs:** including the knowledge needs, highlighting the demand for specific information on managing T1DM in the context of sexual and reproductive health., including sexual activity and childbirth. | romantic and sexual relationships.<br>The need for comprehensive and practical information about T1DM's impact on relationships and sexual health is evident. Emerging adults with T1DM seek knowledge not only about managing the physical aspects of the disease during sexual activities but also about how T1DM intersects with broader aspects of sexual and reproductive health, including libido and pregnancy planning. | |
| 31 | **Helpful aspects of social relationships:** 'giving reminders,' 'instrumental support,' and 'acceptance/emotional support.' These encompassed behaviors such as partners reminding about insulin intake, assisting with diabetes management tasks, and providing a supportive environment for managing diabetes.<br>**Unhelpful aspects of social relationships:** 'Social context barriers' and 'uninformed or misguided others.' These included difficulties faced in managing diabetes due | **Social support dynamics:** Emerging adults combine the themes of helpful and unhelpful aspects of social relationships, highlighting the contrast between supportive actions and barriers created by social environments and uninformed individuals.<br><br>**Role of disclosure in managing social interactions:** The influence of disclosure on the quality of social support, revealing how sharing personal diabetes | Supportive behaviors, such as reminders, instrumental help, and emotional support, significantly ease diabetes management, whereas unpredictable schedules and interactions with uninformed individuals create barriers. Disclosure of diabetes information is pivotal in shaping these social dynamics. Open communication about one's diabetes needs can enhance support from others, whereas a lack of disclosure or presence in settings with uninformed individuals often leads to misunderstandings and unhelpful interactions. | Perceptions, Self-Care, Stigma and Social Relationships: family, peers and partner. |

| | | | | |
|---|---|---|---|---|
| | to unpredictable work or school schedules and challenges posed by people who lack understanding of diabetes (emerging adults anticipated possible stigma).<br>**Factors influencing social relationship dynamics:** disclosure emerged as a key factor. Disclosing diabetes information led to increased helpfulness, whereas a lack of disclosure or presence in unstructured environments with uninformed individuals contributed to unhelpfulness. | information shapes the helpfulness of others. | | |
| 32 | **Disclosure as a measured process**<br>emerging adults described disclosure of their T1DM as a calculated and selective process. They tended to assess their social environment and decide whom to inform based on anticipated reactions and the need for support. This strategic approach to disclosure aimed to balance the need for safety and support with the desire for normalcy and avoiding stigma. Gender-based differences emerged in approaches to disclosure. Men often claimed to be open about their T1DM but displayed behaviors that suggested a preference for privacy. In contrast, women were more likely to disclose for relational reasons, viewing it as an act of trust and responsibility.<br>**The need for lived experience for true understanding:** A recurring sentiment was that only those with T1DM could fully | **Complexities of living with type 1 diabetes in emerging adulthood:** Disclosure is not just a practical matter but also deeply tied to identity, gender dynamics, and personal development. The experience of managing T1DM involves constant self-reflection and adaptation to social environments. | Disclosure is a critical part of emerging adults' navigation in social world but is far from straightforward. It is influenced by a desire to maintain normalcy and independence while acknowledging the need for safety and support. This nuanced approach reflects an adaptive strategy to manage a chronic condition within the social complexities of emerging adulthood. Gender differences in disclosure strategies further underscore the personalized nature of managing T1DM in social contexts. | Emotion al Feelings, Perceptions, Self-Care, Stigma and Social-Relationships: family, peers, partners and work/class-mates. |

| | | | | |
|---|---|---|---|---|
| | understand the intricacies of living with the condition. Emerging adults felt that friends and family, despite their support, lacked the lived experience necessary for true empathetic understanding.<br>**Personal growth and self-awareness:** Living with T1DM leads to a heightened sense of self-awareness and personal growth. Participants recognize the condition as a significant part of their identity and character development, influencing their daily thoughts and actions. | | | |
| 33 | **Interpersonal protective factors:** Emerging adults highlighted the importance of both tangible and emotional support from family, friends, healthcare providers, and community members.<br>Medical and community support played a significant role in providing direction and a sense of care.<br>**Cognitive protective factors:**<br>Emerging adults employed cognitive strategies like normalizing diabetes management as a part of daily life, benefit finding referred to identifying positive outcomes from the experience of living with diabetes, such as personal growth and resilience, and acknowledging the seriousness of their condition, encouraged diligent self-care and management.<br>**Behavioral protective factors:** Strategies for | **Support systems:** Combining interpersonal and some aspects of cognitive factors, the role of family, friends, healthcare providers, and community support emerges as pivotal. This encompasses both the tangible assistance with management tasks and emotional support for coping with diabetes-related challenges.<br>**Personal coping strategies:** Emerging cognitive and behavioral strategies, this category highlights how individuals with T1DM employ personal methods to manage their condition. This includes cognitive approaches like normalization and benefit-finding, and behavioral tactics such as routine establishment, flexibility in management, and the | The support from a network of family, friends, and healthcare providers is critical, providing both practical assistance and emotional support. Cognitive strategies, such as integrating diabetes care into daily life and finding positive aspects in their experiences, enhance resilience and self-efficacy. Behavioral strategies, including maintaining consistency in care, being prepared for emergencies, and leveraging technology, are instrumental in effective diabetes management. These factors collectively facilitate a comprehensive approach to managing T1DM, highlighting the importance of a supportive environment, adaptive mindset, and initiative-taking behaviors in the daily lives of these emerging adults with T1DM. | Emotions and Feelings, Perceptions, Self-Care, Social Relationships: family, peers, peers with T1DM, and healthcare providers. |

| | | | | |
|---|---|---|---|---|
| | successful diabetes management included preparedness (involved planning for diabetes urgent needs or emergencies and carrying necessary supplies), consistency (maintained through regular routines in diabetes management), adaptability (adjusting plans to accommodate unexpected diabetes management needs), balance (involved managing diabetes while also engaging in non-diabetes related activities), and the use of technology, such as continuous glucose monitors, insulin pumps, and smartphone apps, facilitated diabetes management. | adoption of technology aids for diabetes care. | | |
| 34 | **Sensitivity to perceptions of other without diabetes:** Emerging adults exhibited heightened awareness and concern regarding how they are perceived by those without T1DM. The presence of T1DM technology, often instigated self-consciousness and anxiety, particularly around physical appearance, and diabetes management efficiency.<br>**Boundaries and independence:** There was a diverse spectrum of boundary-setting with romantic partners concerning T1DM. While some participants preferred to keep their glycemic levels private to maintain independence, others found value in sharing this information for support and safety.<br>**Support from partners:** Support from | **Interpersonal dynamics in T1DM:** this includes the dynamics of disclosure, the role of health technology in these relationships, and the balance between independence and support in T1DM management within romantic partnerships.<br>**Emotional and practical aspects of T1DM management:** The emotional burden of living with T1DM, heightened by technology visibility and partner interactions, intersects with the practical aspects of T1DM management. This includes the strategic disclosure of T1DM status, sensitivity to partner reactions, and reliance on technological aids for health monitoring and | The role of romantic partners emerges as both a source of support and a factor in determining the level of privacy and independence desired in managing T1DM. Health technology, while providing critical monitoring and management tools, also brings an added layer of emotional complexity, influencing how individuals with T1DM navigate disclosure, self-perception, and independence. | Emotion and Feelings, Perceptions, Social Relationships: partners. |

| | | |
|---|---|---|
| | romantic partners was generally seen as positive, with partners providing both emotional and practical assistance. Instances where partners were directly involved in T1DM management, such as responding to low blood sugar alerts, were particularly valued. | emergency alerts. |

Abbreviations
T1DM: Type 1 Diabetes Mellitus
