# Supplementary material for: Lifestyle in Emerging Adults with Type 1 Diabetes Mellitus: A Qualitative Systematic Review
Source: Healthcare (Basel). 2024 Jan 25;12(3):309. doi: 10.3390/healthcare12030309 (PMC10855310; doi:10.3390/healthcare12030309)
Supplement: Supplementary file 1 [file healthcare-12-00309-s001.zip › Supplementary Material Table S2- ConQualR2_Revised.pdf]

Supplementary Material Table S2 – CONQual

Table S2. Confidence in the findings: Application of CONQual.

	SYNTHESIZED FINDINGS	DEPENDABILITY	CREDIBILITY	CONQUAL SCORE
1	Transition to adulthood for those people managing diabetes involves significant changes in family support and self-care responsibilities. This phase sees a shift from parental guidance to greater self-management, promoting individual autonomy. Individuals adapt their self-identity, integrating the condition into their self-understanding. Influenced by changing relationships and social dynamics, this adaptation leads to an ongoing reassessment of how they choose to disclose or conceal their condition in social contexts.	Moderate	Unequivocal	Moderate
2	A complex interplay of emotional, psychological, and practical challenges faced by emerging adults living with T1DM. Emotional distress and societal perceptions significantly impact their diabetes self-management practices. These challenges, coupled with the complexities of navigating healthcare systems and daily routines, underscore the heightened risk of both acute and long-term diabetes-related complications. The effectiveness of self-management is influenced by each individual's physical environment, healthcare experiences, and ability to integrate diabetes care into daily life, all of which are crucial in mitigating these risks.	High	Unequivocal and Credible findings	Moderate
3	The multifaceted challenges faced by individuals in managing diabetes clinic appointments. Systemic barriers such as inefficient communication and rigid clinic hours compound personal struggles, including dealing with unsympathetic employers and negative interactions with healthcare professionals. These factors collectively influence patients' decisions regarding clinic attendance, often leading to a complex pattern of engagement with healthcare services. Emotional support and practical information are highly valued by patients, but negative experiences can significantly deter future attendance, indicating a need for more patient-centric and flexible healthcare approaches.	Moderate	Unequivocal	Moderate
4	A complex interplay between diabetes management, body image, and eating disorders among young adults. Females expressed significant concerns about weight gain linked to insulin use, leading to dangerous practices like insulin omission. This behaviour often started inadvertently but escalated into more severe eating disorders, profoundly impacting their health and daily life. The findings also highlight systemic shortcomings in healthcare and the varied role of family support, pointing to a need for more comprehensive and sensitive approaches to address these intertwined issues.	Moderate	Unequivocal and Credible findings	Low
5	The study uncovers a complex array of factors influencing diabetes management adherence among young adults. A collaborative approach by healthcare providers, along with supportive social networks, significantly boosts	Moderate	Unequivocal	Moderate

	engagement. Conversely, non-engagement is often driven by psychological factors such as treatment fatigue and self-efficacy issues, as well as systemic challenges like varying healthcare experiences and lack of consistent support. These findings emphasize the need for personalized and empathetic healthcare strategies that address the diverse needs and challenges faced by individuals with diabetes.			
6	Significant challenges emerging adults with diabetes face in managing their condition within demanding and non-routine work environments. Time pressures, workplace stress, and the need to conform to workplace norms often lead to compromised diabetes management. These findings highlight the necessity for flexible and adaptable diabetes care strategies that can be integrated into diverse and dynamic work schedules, as well as the importance of supportive workplace policies and practices.	Moderate	Unequivocal	Moderate
7	Emerging adults with T1DM often face an unstructured and challenging transition from pediatric to adult care. This transition is characterized by a lack of preparation and guidance, leading to vulnerabilities during college years and surprise at the differences in adult care. Participants express a desire for a more intentional and supported transition, with specific guidance and peer support to adapt to the new reality of adult diabetes care.	High	Unequivocal	High
8	A critical gap in structured transition support for emerging adults with T1DM. The participants' experiences highlight the complexities of navigating the healthcare system, along with developmental and psychosocial factors (sense of invincibility) that complicate diabetes management during this transition phase. These findings emphasize the need for targeted interventions (risk behaviors) and enhanced communication strategies to facilitate a smoother transition from pediatric to adult healthcare for emerging adults with T1DM.	High	Unequivocal	High
9	Multifaceted challenges faced by emerging adults with T1DM, encompassing emotional, social, environmental, and healthcare-related factors. These challenges often interplay, creating a complex scenario that hampers effective diabetes management. Emotional struggles, combined with social pressures and inadequate support systems, lead to poor adherence to treatment and lifestyle modifications. The findings call for a more integrated approach in diabetes care, addressing not only the medical aspects but also the emotional, social, and psychological needs of emerging adults.	Moderate	Unequivocal	Moderate
10	The challenges of maintaining blood glucose levels in range, coupled with the psychological and social aspects of living with diabetes, underscore the need for comprehensive support systems. Accepting one’s condition and integrating it into their identity is a crucial part of this journey. These insights suggest a need for personalized care strategies that address not only the medical aspects but also the emotional, social, and practical challenges faced by emerging adults with T1DM.	High	Unequivocal	High
11	The complex nature of managing T1DM among young women, highlighting the elusive nature of maintaining glycaemic stability and the significant role of technology in facilitating management. It underscores the need for adaptable routines and strategies to deal with the unpredictable nature of diabetes, particularly in relation to lifestyle	High	Unequivocal and credible findings	Moderate

	changes, exercise, and menstrual cycles. Emotional and psychological challenges are prevalent, stemming from the fluctuating nature of the disease, weight management issues, and interactions with healthcare providers. Ultimately, these experiences converge into the central theme of seeking balance, underscoring the need for holistic approaches in diabetes care that consider both the physical and emotional aspects of the condition.			
12	The complexities of managing T1DM during the transition to college life. Effective management requires meticulous planning and adaptation to new routines. The participants' ability to maintain a positive outlook, despite the challenges, illustrates resilience and an initiative-taking approach to health. The role of support systems, both formal (diabetes organizations) and informal (family and friends), is pivotal in providing the necessary guidance, emotional support, and sense of community. These findings underscore the need for tailored support strategies to assist emerging adults with T1DM in successfully navigating this critical life transition.	High	Unequivocal and credible findings	Moderate
13	The multifaceted experiences of emerging Appalachian adults with T1DM. It underscores the intricate balance between the challenges of managing T1DM and the transition into adulthood. The findings reveal how T1DM significantly shapes personal identity and daily life, posing both physical and psychosocial challenges. Despite these difficulties, there is a notable resilience and positivity among participants, supported by strong familial, social, and healthcare networks. The role of technology emerges as a double-edged sword, offering enhanced management tools yet creating new dependencies. Overall, these insights underscore the need for holistic support systems that address both the medical and psychosocial aspects of living with T1DM in emerging adulthood.	High	Unequivocal	High
14	The complex interplay between the practical and psychological aspects of managing T1DM in emerging adult women. The desire for freedom and normalcy conflicts with the ever-present nature of diabetes management. Despite integrating diabetes into their identity, these women grapple with the visibility of their condition and its impact on self-perception. The findings underscore the need for support systems that address not only the physical but also the emotional and social challenges of living with T1DM.	Moderate	Unequivocal	Moderate
15	A key aspect is the continuity of medical care from pediatric to adult providers, accompanied by logistical challenges in scheduling and traveling for appointments. The positive impact of specialized diabetes healthcare professionals' contrast with the general lack of diabetes-specific knowledge and resources on college campuses. Students suggest the need for more tailored resources, such as on-campus diabetes specialists.<br>Social support emerges as a vital element, with friendships providing emotional and practical support. Particularly, connections with peers who also have diabetes offer a unique understanding and empathy. This social network is instrumental in helping students navigate the complexities of diabetes management within the dynamic college environment.	Moderate	Unequivocal and Credible findings	Low
16	Successful healthcare transition for emerging adults T1DM is marked by a multifaceted integration of autonomy and	High	Unequivocal	Moderate

	proficiency in disease management. Those who navigate this transition adeptly maintain biomedical levels, as evidenced by appropriate HbA1C levels, and exhibit effective self-management skills. Navigating the adult healthcare system involves establishing collaborative relationships with healthcare providers and striking a balance between parental involvement and independent self-care. A unifying element across these aspects is the attainment of 'ownership' of the disease, where the individual acknowledges T1DM as an integral part of their identity and actively assumes responsibility for their care. In summary, a successful transition is multidimensional, blending medical, personal, and supportive elements to foster independent and effective diabetes management in adulthood.		and Credible findings	
17	There is a significant gap in terms of information and interventions designed for emerging adults with type 1 diabetes. This is due to the healthcare system traditionally focusing on childhood and adolescence for type 1 diabetes, and on type 2 diabetes during adulthood. There are psychological and physical challenges in this population, which reports experiencing stigmatization from society and calls for interventions tailored to lifestyle changes and the adoption of stereotypical behaviours such as alcohol consumption, as well as the support of mental health care and social support.	High	Unequivocal	High
18	Self-care motivation in emerging adults with T1DM is multifaceted, influenced by both internal and external factors. The presence of a supportive environment, management of one's life, and a sense of wellbeing significantly bolster self-care motivation. In contrast, feelings of vulnerability and exclusion, often due to societal misconceptions and pressures, pose substantial barriers to consistent self-care. These findings suggest a need for a holistic approach to emerging adults with T1DM	High	Unequivocal and credible findings	Moderate
19	Effective diabetes management in emerging adults' hinges on a composite support system encompassing family, friends, and healthcare professionals. Familial support -primarily emotional, practical, and financial- is vital in fostering companionship and managing daily challenges. Healthcare providers offer crucial informational support, enhancing understanding and management of diabetes management. Friends augment this support by providing both practical assistance and emotional solidarity, demonstrating the importance of a well-informed social network. The collaboration of family, peers, and healthcare professionals creates a dynamic support framework, addressing the diverse needs of diabetes management. This research highlights how awareness and education within one's social circle contribute to effective diabetes care and emotional well-being, reinforcing the idea that a multi-dimensional support network is essential for navigating the complexities of diabetes management in emerging adults.	Moderate	Unequivocal	Moderate
20	A new identity that incorporates diabetes management as an integral part. This process involves a significant degree of personal empowerment, as individuals learn to make informed decisions about their health and lifestyle. The role of social support, encompassing family, friends, and healthcare providers, emerges as a cornerstone in their life, offering necessary practical and emotional sustenance. Moreover, the findings reflect the participants' resilience in adapting their life goals and aspirations to accommodate their condition.	Moderate	Unequivocal	Moderate

21	Emerging adults with T1DM face complex challenges in managing both their condition and weight. They navigate hypoglycemia as a barrier to weight management, confront societal stigmas, and express a need for personalized diabetes technology. Transitioning to adulthood introduces new dimensions: romantic relationships, financial burdens, and work commitments significantly impact T1DM and weight management. In emerging adulthood, specific challenges emerge. Concerns about family planning and fertility, alongside evolving relationships with medical providers, highlight the unique struggles in this life stage. These findings underscore the need for nuanced support as emerging adults balance T1DM management with the intricacies of adult life.	Moderate	Unequivocal	Moderate
22	Emerging adulthood is marked by an intense sense of autonomy and a desire of managing their condition, counterbalanced by the emotional toll of constant vigilance. The supportive role of healthcare professionals is highly valued, providing a contrast to the challenges faced in social settings, particularly in managing the impacts of alcohol on their condition. The social aspect of living with diabetes is multifaceted, characterized by a mixture of support and misunderstanding from others. A recurrent theme is the struggle with sustaining motivation for continuous self-care, reflecting the ongoing challenge of balancing diabetes management with the desire for a normal life.	Moderate	Unequivocal	Moderate
23	The multifaceted challenges faced by emerging adults with T1DM with managing their condition amidst the pressures, schedules, and lifestyles of college life. The interplay of immediate diabetes management challenges with longer-term worries about health and life implications is a persistent theme. Additionally, the critical role of social networks in emergency situations underscores the importance of community awareness and support in managing chronic conditions like T1DM.	High	Unequivocal and credible findings	Moderate
24	There is a spectrum of experiences among emerging adults with T1DM, characterized by varied narrative lenses. The ingrained lens indicates a successful integration of diabetes into one's life, whereas the intrusive lens reflects ongoing struggles and burdens. The inconspicuous lens suggests a tendency to minimize the impact of diabetes, possibly as a coping mechanism.	High	Unequivocal	High
25	Daily life activities are complicated by the need for constant management and vigilance, impacting both physical and emotional well-being. Participants experience a tension between managing their condition privately and the public exposure of their self-care practices, often leading to uncomfortable social interactions and public scrutiny. Misconceptions about diabetes, especially the confusion between T1DM and T2DM, contribute to a stigma that affects how individuals with T1DM are perceived and treated. This misunderstanding extends to health moralism, where participants face judgment about their lifestyle choices that are governed by the necessities of managing T1DM.<br>This comprehensive view underscores the need for greater public awareness and understanding of T1DM and its impact on individuals' lives. It highlights the importance of support systems that recognize the emotional and social challenges of living with T1DM, in addition to the physical aspects of the condition.	High	Unequivocal and credible findings	Moderate

26	The transition to greater independence brings new complexities in managing diabetes, where finding a balance between daily life and diabetes care is a constant struggle. Participants want to manage their condition, yet this stability is often hindered by the relentless demands of diabetes management. The hidden burden of diabetes is a significant theme, where managing the condition is an internal, often private struggle that is not always apparent to others. This leads to challenges in social situations and a desire to maintain normalcy without drawing attention to their condition. The relationship with diabetes providers is pivotal. Participants desire a partnership approach, where providers are not just addressing medical needs but also understanding the life challenges and context of each individual. This is a holistic approach to effective diabetes management and overall well-being.	High	Unequivocal and credible findings	Moderate
27	T1DM affects all aspects of emerging adults' lives, from academics to social interactions, requiring a constant balancing act. The unique college environment poses specific challenges for diabetes management, influencing food choices, physical activity, sleep, and overall routine. The experience of living with T1DM in college is not only a challenge but also an opportunity for growth. Emerging adults report accelerated maturity, increased responsibility, and a changed outlook on life. They navigate their condition using a variety of strategies, from seeking support networks to actively learning through trial and error. Self-advocacy emerges as a crucial skill, especially in negotiating the college environment and managing health The college should be a tailored support system for students with T1DM. It highlights the importance of understanding the unique challenges faced in the college setting and the need for strategies that support both diabetes management and personal development.	Moderate	Unequivocal	Moderate
28	Poor sleep hygiene, stress, and an uncomfortable sleep environment are general barriers to obtaining sufficient sleep. In the context of diabetes, managing blood glucose levels and equipment at bedtime poses additional challenges, with the fear of hypoglycemia being a significant concern.<br>To facilitate better sleep, emerging adults adopt various strategies. These include establishing a regular bedtime routine, engaging in physical activities during the day, creating a comfortable sleep environment, and using relaxation techniques such as meditation or light exercise. For diabetes-specific facilitators, maintaining blood glucose within a desired range before bedtime is critical. This involves careful meal planning, snack management, and effective use of diabetes equipment.	High	Unequivocal	High
29	Routine diabetes care activities are often prompted by specific cues and are integral to effective self-management. However, life transitions, such as moving to college, can disrupt these established routines, leading to challenges in maintaining consistent diabetes care. The conduciveness of the environment, level of social support, and presence of diabetes-related stigma can either facilitate or hinder effective diabetes care. Internal factors such as stress and self-reliance also significantly impact how individuals manage their condition.	High	Unequivocal and credible findings	Moderate
30	Relationship dynamics are significantly influenced by T1DM, requiring of open communication and a deep	Moderate	Unequivocal	Moderate

	understanding of the disease within the relationship. This mutual comprehension is vital for navigating the challenges and adjustments that T1DM brings to romantic and sexual relationships. The need for comprehensive and practical information about T1DM's impact on relationships and sexual health is evident. Emerging adults with T1DM seek knowledge not only about managing the physical aspects of the disease during sexual activities but also about how T1DM intersects with broader aspects of sexual and reproductive health, including libido and pregnancy planning.			
31	Supportive behaviors, such as reminders, instrumental help, and emotional support, significantly ease diabetes management, whereas unpredictable schedules and interactions with uninformed individuals create barriers. Disclosure of diabetes information is pivotal in shaping these social dynamics. Open communication about one's diabetes needs can enhance support from others, whereas a lack of disclosure or presence in settings with uninformed individuals often leads to misunderstandings and unhelpful interactions.	High	Unequivocal	High
32	Disclosure is a critical part of emerging adults' navigation in social world but is far from straightforward. It is influenced by a desire to maintain normalcy and independence while acknowledging the need for safety and support. This nuanced approach reflects an adaptive strategy to manage a chronic condition within the social complexities of emerging adulthood. Gender differences in disclosure strategies further underscore the personalized nature of managing T1DM in social contexts.	High	Unequivocal	High
33	The support from a network of family, friends, and healthcare providers is critical, providing both practical assistance and emotional support. Cognitive strategies, such as integrating diabetes care into daily life and finding positive aspects in their experiences, enhance resilience and self-efficacy. Behavioral strategies, including maintaining consistency in care, being prepared for emergencies, and leveraging technology, are instrumental in effective diabetes management. These factors collectively facilitate a comprehensive approach to managing T1DM, highlighting the importance of a supportive environment, adaptive mindset, and initiative-taking behaviors in the daily lives of these emerging adults with T1DM.	High	Unequivocal	High
34	The role of romantic partners emerges as both a source of support and a factor in determining the level of privacy and independence desired in managing T1DM. Health technology, while providing critical monitoring and management tools, also brings an added layer of emotional complexity, influencing how individuals with T1DM navigate disclosure, self-perception, and independence.	High	Unequivocal and credible findings	Moderate

Abbreviations
T1DM: Type 1 Diabetes Mellitus
